# ELM-KL-LSTM: a robust and general incremental learning method for efficient classification of time series data

**DOI:** 10.7717/peerj-cs.1732

**Published:** 2023-12-21

**Authors:** Qiao Zhou, Zhong-Yi Wang, Lan Huang

**Affiliations:** 1 College of Information and Electrical Engineering, China Agricultural University, Beijing, China; 2 Ministry of Agriculture, Key Laboratory of Agricultural Information Acquisition Technology (Beijing), Beijing, China; 3 Ministry of Education, Key Laboratory of Modern Precision Agriculture System Integration Research, Beijing, China

**Keywords:** Concept drift, Lightweight preprocessing model, Model update strategy, Efficient classification analyzing, Dynamically changing time series data

## Abstract

Efficiently analyzing and classifying dynamically changing time series data remains a challenge. The main issue lies in the significant differences in feature distribution that occur between old and new datasets generated constantly due to varying degrees of concept drift, anomalous data, erroneous data, high noise, and other factors. Taking into account the need to balance accuracy and efficiency when the distribution of the dataset changes, we proposed a new robust, generalized incremental learning (IL) model ELM-KL-LSTM. Extreme learning machine (ELM) is used as a lightweight pre-processing model which is updated using the new designed evaluation metrics based on Kullback-Leibler (KL) divergence values to measure the difference in feature distribution within sliding windows. Finally, we implemented efficient processing and classification analysis of dynamically changing time series data based on ELM lightweight pre-processing model, model update strategy and long short-term memory networks (LSTM) classification model. We conducted extensive experiments and comparation analysis based on the proposed method and benchmark methods in several different real application scenarios. Experimental results show that, compared with the benchmark methods, the proposed method exhibits good robustness and generalization in a number of different real-world application scenarios, and can successfully perform model updates and efficient classification analysis of incremental data with varying degrees improvement of classification accuracy. This provides and extends a new means for efficient analysis of dynamically changing time-series data.

## Introduction

Time series data are one of the most common data types in daily life, carrying rich information, which efficient analysis has great value. However, the dynamically changing time series data always have various uncertain characteristics such as different degrees of conceptual drift, anomalous data, erroneous data and even missing data, *etc*. Thus, these will cause great difference of feature distribution changes of new data within a sliding window, which efficient analysis still remains challenges.

At present, the existing research on efficient classification analysis or prediction of time series data mainly includes traditional statistical methods (Box et al., 2015; Rhif et al., 2019), intelligent computing methods (Ademola & Ahmad, 2004; Cao, Li & Li, 2019; Cao & Gu, 2002) and combinatorial methods (Dietterich, 2002; van Heeswijk et al., 2009; Ruping, 2001). The traditional methods are based on the linear structural relationship between data variables and can only mine and extract the shallow linear features, which makes it difficult to achieve deep analysis in practice, moreover, they cannot meet the efficient classification analysis or prediction for the growing dynamic changing time series data. In contrast, the intelligent computing methods are based on the powerful machine learning models and deep learning models (Jogin et al., 2018; Lafabregue et al., 2022; Dara & Tumma, 2018), *etc*., and are better at mining the dataset’s deep nonlinear feature information, which makes them very successful. However, nowadays the obvious fact is that when facing a highly uncertain and dynamically changing dataset like the growing and constantly updated time-series data, a single model or method is somewhat overwhelmed with its robustness, generalization and model performance decreasing or even failing to accurately capture the data distribution and changing patterns, while retraining the model often requires high costs, not to mention its inability to meet efficient tasks. Compared to intelligent computational methods, the current combinatorial approach demonstrates its good superiority and flexibility by fusing several different models and strategies (including traditional and intelligent computational methods) (Salles et al., 2022; Arik, Yoder & Pfister, 2022) to obtain more accurate and comprehensive deep nonlinear representation information of the dataset, thus improving the overall model efficiency and ultimate performance. Among which, IL and Ensemble Learning (EL)-based approaches for efficient analysis of time series data are hot research topics (Dong et al., 2020; He et al., 2020; Sagi & Rokach, 2018; Wu et al., 2019). In fact, the IL based methods show greater flexibility and efficiency (Ade & Deshmukh, 2013; Masana et al., 2022; Zhou et al., 2023). Therefore, in order to achieve efficient classification analysis of dynamically changing time series data while balancing classification accuracy and efficiency, we proposed a new method called ELM-KL-LSTM, which combines the idea of incremental learning, ELM, and deep learning. We conducted extensive verification and analysis based on real-world important multi-scenario, and the experimental results show that the proposed method exhibits good robustness, generalization, successful model update and efficiency analysis for incremental data in several different real-world application scenarios. This provides and extends a new means for efficient analysis of dynamically changing time series data. The contributions of this article are as follows.

(1) For efficient classification analysis of dynamically changing time series data, we proposed a new incremental learning based method ELM-KL-LSTM.

(2) For the sake of eliminating the large variation of the dataset feature distribution difference to a certain extent due to various degrees of concept drift, anomalous data, erroneous data and high noise in the newly generated dataset, and improve the information representation, accuracy and inter-sample variability, we proposed a lightweight pre-processing model based on ELM.

(3) To develop a reasonable and effective lightweight preprocessing model initiation mechanism, we measured the degree of feature distribution differences between old and new datasets based on KL scatter values, developed the evaluation metrics and designed the model update strategy.

(4) We conducted an extensive study and comparation analysis based on the proposed method and benchmark methods in several different real application scenarios.

Especially, this is the first efficient classification application study when facing the dynamically changing time series data in the field of plant electrophysiology, which provides a new effective method for future research on rapid stress resistance breeding, plant electrophysiology and the related research (Qin et al., 2020; Wang, Fan & Qian, 2018; Wang et al., 2019; Yao et al., 2021), and has great significance.

The rest of this article is organized as follows. “Related Works” provides a brief summary of the ELM or LSTM based incremental learning for time series datasets. “The Proposed Method” introduces the details of the proposed model. “Experiments and Analyses” presents the experimental results and evaluations for proposed method under multiple scenarios, and then discusses the advantages and limitations. “Conclusion” concludes the article in final.

## Related works

This section first reviews the ELM and LSTM based incremental learning studies for time series data, and then discusses our ideas and motivations for doing this work.

### The ELM based data analysis

Due to its good robustness, generalization and extremely fast learning ability, the ELM has shown great potential in applications and gained high attention from academia and industry, which has been widely used and achieved fruitful results in disease diagnosis, emotion recognition, fault diagnosis, biomedicine, computer vision, *etc*., (Deng et al., 2016; Liang et al., 2006; Liu, Wu & Jiang, 2016; Zhao, Wang & Park, 2012). For time series prediction, Salles et al. (2022) was the first to propose an integrated framework ESPred which integrate multiple data transformation methods, advanced data statistics and machine learning methods, based on which users can build the desired models by themselves, which also includes ELM and LSTM as prediction models, respectively, but does not address the dynamically changing datasets and model update problem. For solving the financial time series forecasting problem, Huihui & Qun (2021) proposed an adaptive incremental ensemble learning (SIEL) algorithm with ELM as the base model while does not have more application in multi-scenario. Combining incremental learning and ELM, Xue et al. (2018) proposed the incremental multiple kernel (IMK)-ELM algorithm and applied it to intelligent financial recommendation systems. For online sequences, Liang et al. (2006) proposed an online sequential (OS)-ELM model, which first initializes the output weights of the network with a small number of training samples, and then acquires good online learning capability in the process of incremental learning. Deng et al. (2016) proposed an online sequential reduced kernel ELM OS-RKELM algorithm to provide a unified learning framework for classification and regression tasks in online incremental learning. Based on multiple ELM models (Liu & Wang, 2010) proposed an ensemble based ELM algorithm (EN-ELM), which introduces EL methods and cross-validation strategies into the training process to mitigate overfitting and improve model stability and classification accuracy. There are also many other related studies such as van Heeswijk et al. (2009), Liu, Wu & Jiang (2016), Mirza, Lin & Toh (2013), Wu et al. (2016), Yu & Dai (2021), ECG signals can be recognized effectively ELM-based user identification, which improves the recognition accuracy and realizes online incremental data learning to a certain extent (Chen et al., 2023). A fully online sequential extreme learning machine (FOS-ELM) model was proposed in Chupong, Junhuathon & Plangklang (2022), which can learn incrementally without the need for initial training samples and is suitable for application where historical load data is not available to train the model. ELM was studied and evaluated as an incremental learning network in Abramova Elena, Orlov Alexey & Makarov Kirill (2022). It was pointed out that extreme learning machine (ELM) has incremental learning ability and faster learning rate, and which allows neural network to adapt to new data while retaining existing data. Facing the characteristics of dynamic changes of intrusion types in the industrial Internet of things, a real-time network intrusion detection system based on ELM is proposed, and the computational efficiency is improved (Gyamfi & Jurcut, 2022). A novel specific emitter identification (SEI) scheme based on ELM and variational mode decomposition is proposed in Zhang et al. (2023), which effectively reduces the training cost and improves the identification accuracy. There are many researches on time series data analysis based on ELM, most of which use its efficient computing power.

To sum up the above existing studies, most of them are for a specific application scenario or only static or dynamic time series data. The advantages of the study on the dynamic time series based on ELM are flexibility and high efficiency. However, because of the extremely simple network structure of ELM, it has limitations in the deep spatial-temporal correlation information extraction (Alom et al., 2019; Huang et al., 2015; Wang et al., 2022). Therefore, it is difficult to realize efficient in-depth analysis with good performance only depending on ELM. For efficient deep analysis of dynamically changing time series data, combining the high efficiency of ELM with the deep information representation ability of deep learning model is an interesting research direction.

### LSTM based incremental learning

As one of the most classical methods of deep learning, LSTM is an extremely typical recurrent neural network with temporal memory characteristics, which has natural advantages in processing time series data, and becomes one of the most common, powerful and classical tools in time series data applications and a research hotspot (Smagulova & James, 2019; Yu et al., 2019). There have been many related studies based on LSTM. Among them, to address the problem of insufficient information mining of emotional features and low recognition accuracy in EEG signal emotion recognition, Peng & Yan (2022) proposed a deep autoencoding method to extract emotional features of EEG signals. This method was combined with LSTM to achieve multidimensional emotional classification. A multi-scale convolution and attention mechanism (MCA)-based LSTM model (MCA-LSTM) was proposed in Xuan, Wan & Chen (2022), which can focus on and fuse important multi-scale features to achieve more accurate classification. For the problem of financial time series predicting, a new ensemble model based on LSTM networks was proposed in Preeti, Rajni & Singh (2019), which can extract nonlinear nature of time series. For the prediction of dynamically changing time series data, Wang, Li & Yue (2021) proposed an incremental learning ensemble model INcLSTM with LSTM as the base model, which to some extent solves the problem that the offline model needs to be constantly updated and improves the efficiency of the model, but the application scenario is relatively single. Based on BiLSTM and attention mechanism, Zhao et al. (2022) predicted the remaining service life of engines and bearings, but there is no application involving many other dynamically changing time series data. In addition, based on the other deep learning models, there are also many studies on efficient classification analysis of time series data, such as convolutional neural networks (Rostamian & O’hara, 2022; Zeng, Xiao & Zhang, 2016; Zhao et al., 2021), Siamese neural networks (Malialis, Panayiotou & Polycarpou, 2022). Among which, for efficient classification of time series data, Malialis, Panayiotou & Polycarpou (2022) proposed an incremental learning method ActiSiamese based on active learning and siamese neural networks which can solve the problem of non-equilibrium appearing to some extent. Based on LSTM and DNN, a new method which can be used for network intrusion detection is proposed in Han & Pak (2023), greatly reducing detection delay and achieving high detection performance, which is of significant help to improve network security performance. Facing the dynamically changing 3D attitude data, an incremental learning method based on LSTM is proposed for real-time 3D attitude estimation, which solves the uncertainty and unpredictability of dynamic data to a certain extent (Narkhede et al., 2021). Aiming at the dynamic changing data stream, an incremental learning method based on the variation of the original LSTM network is proposed, which can solve the concept drift and model generalization to a certain extent and improve the computational efficiency (Lemos Neto, Coelho & de Castro, 2022). Aiming at the emotional classification of users on product evaluation in e-commerce websites, a two-stage mixed incremental learning method based on LSTM and SVM is proposed in Londhe & Rao (2022), which effectively improves the recognition accuracy. Aiming at the classification problem of dynamically increasing online information flow in social media, a classification method based on incremental learning is proposed based on LSTM and ELM, in which ELM implements incremental learning and LSTM implements classification analysis. This model has certain generalization (Jaganathan & Madhusudhanan, 2022). For atrial fibrillation detection, an incremental learning method is proposed based on transfer learning and active learning which combined several deep neural networks such as convolutional neural network and LSTM network, realizing the effective analysis of Atrial fibrillation data along with the model updating strategy (Shi et al., 2020).

Based on the abovementioned literature analysis, we can conclude that although the studies on LSTM-based incremental learning have demonstrated good performance and effectiveness in various fields, the majority of these studies are focused on specific applications (Alade, Selamat & Sallehuddin, 2018; Wang et al., 2022), and the time required for model updates remains a challenge. If all incremental learning methods are based on deep learning models, their performance, especially in terms of efficiency and flexibility, is still limited by their inherent characteristics such as large model size. Therefore, to further improve efficiency and flexibility, it is necessary to find more efficient and flexible processing models, and ELM can meet these requirements. Therefore, combining the high efficiency of ELM with the deep information representation ability of LSTM is a promising research direction for efficient deep analysis of dynamically changing time series data.

### Analysis and motivation

From the above statements, we see that ELM has the simple structure, good robustness, generalization and very fast learning ability with great potential. However, it is deficient in deep feature extraction and deep information representation compared to deep learning. In contrast, as a kind of deep learning, LSTM has a natural advantage in processing time series data, and can selectively extract deep nonlinear features with correlations before and after the time series. Therefore, we proposed an ingenious computational framework to achieve a balance between efficiency and accuracy.

## The proposed method

### Overview of the proposed method

**Figure 1:**
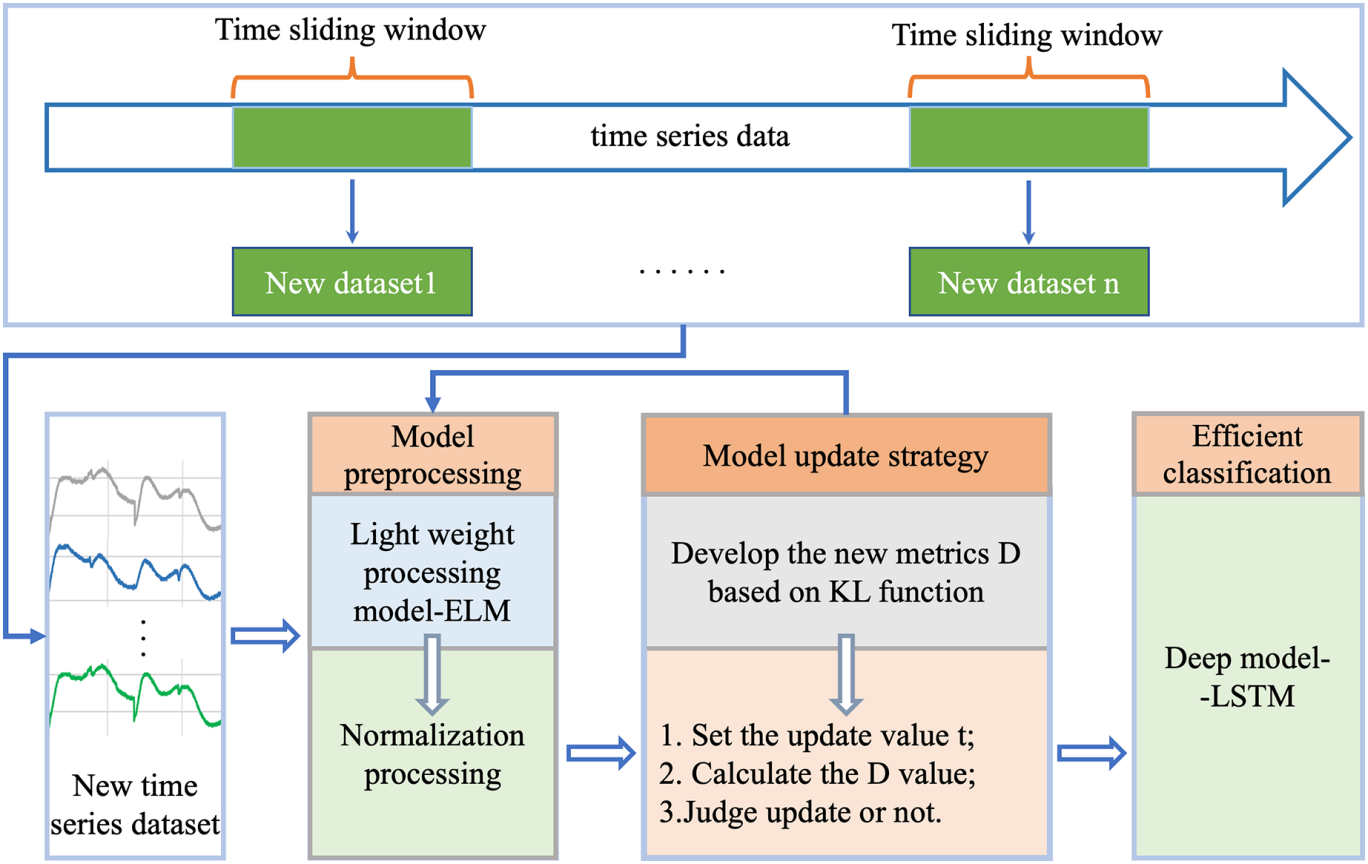
Overall flow of the proposed framework of ELM-KL-LSTM.

For efficient classification analysis of dynamically changing time series data, we proposed a new robust generalized incremental learning method ELM-KL-LSTM based on ELM and LSTM, as shown in Fig. 1, which mainly consists of four parts: 1. new datasets update; 2. model preprocessing; 3. model update strategy; 4. efficient classification model. Part one is to continuously acquire new and different time series datasets; the lightweight model preprocessing is based on the model update strategy to decide whether to preprocess the updated datasets, herein we designed the lightweight preprocessing model based on ELM which has certain potential in processing datasets with insignificant changes in feature distribution. The model update strategy is based on our designed evaluation metrics D which can measure the difference degree in feature distribution between the new and the old dataset to determine whether the lightweight preprocessing model is started. As the final classification model, the LSTM based model is trained in advance based on the existing dataset and is responsible for the efficient classification computation on the new datasets at any time. The function and details of each part are described in the following sections.

### Incremental learning model

#### New datasets update

In this article, we conducted experiments and analysis based on five important real-world application scenarios, which will be described in “Datasets and Data Preprocessing”. To continuously acquire new datasets of time series data, we set parameters such as update window size, number of windows, update steps, *etc*., and decide to update the sample or feature according to the characteristics of each dataset such as sample size, waveform periodicity, waveform length and feature variability. In this article, the dataset update strategy is mainly as follows: 1. If the sample size of the dataset is small, the number of sample feature points is sufficient and the waveform has a certain periodicity, the feature is updated; for example, assuming that the sample length is L and its periodic wave length is w, we generally set the update window size to w * n (n ≥ 1) in combination with its periodicity, and the number of windows N needs to meet w * n * N ≤ L. The update step is generally taken a cycle wavelength w. 2. If the dataset is rich enough in samples size, while its sample feature points are not so many and the waveform does not have periodicity, the sample is updated. The specific update settings of each dataset used in this article will be presented in “Experimental Results”.

#### Model pre-processing

**Figure 2:**
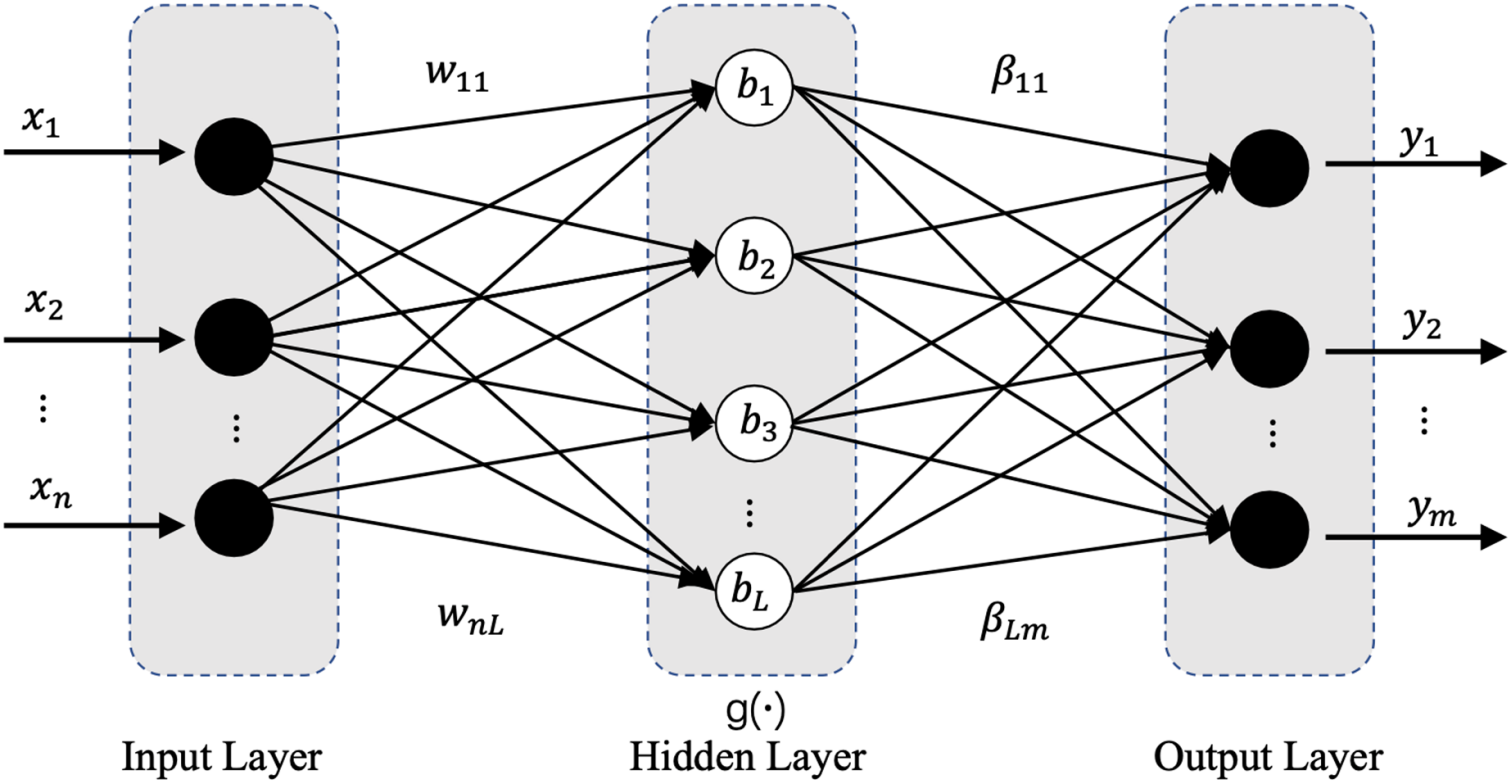
Network structure diagram of ELM.

Model preprocessing consists of two parts, lightweight model preprocessing and normalized preprocessing. In this work, we designed a lightweight preprocessing ELM-based model. ELM has a powerful computing ability with less human intervention (Liang et al., 2006), more importantly, its current combination with deep learning has shown great potential and achieved wide attention and rich results. The ELM based studies have shown which certain potential in processing datasets with large feature distribution differences (Bayram, Ahmed & Kassler, 2022; Huang, Zhu & Siew, 2006; Xu & Wang, 2017; Yang et al., 2019). The ELM’s network structure is extremely simple with only three network layers: input layer, hidden layer, and output layer, and its basic network structure is shown in Fig. 2. In our applied ELM network, the parameters are set as follows: input_nums = 5, hidden_nums = 32, output_nums = 1, and the activation functions are Sigmoid. The main task of the lightweight model is when the difference in feature distribution between the old and new datasets is greater than the update threshold, the ELM based model will start rapidly in real time and pre-process the samples of the new datasets through its efficient and powerful regression function, which will perform a certain degree of weakening, removal, correction and optimization for the different degrees of conceptual drift, anomalous data, erroneous data and even missing data occurring in the newly generated datasets, and improve the information representation, accuracy and inter-sample variability, and finally obtain a new dataset which similar or consistent with the old dataset in terms of feature distribution. The standardized preprocessing is to improve the computational efficiency and accuracy of the following efficient classification model which described in “Evaluation Metrics”.

The mathematical model of ELM network is as follows:

(1) $$\matrix{ {Y = \; {f_L}\left( x \right) = \; \mathop \sum \nolimits_{i = 1}^L {\beta _i}{h_i}\left( x \right) = h\left( x \right)\beta } \cr }$$

(2) $$\matrix{ {h\left( x \right) = \; \mathop \sum \nolimits_{i = 1}^L g\left( {{w_i}.x + {\rm \; }{b_i}} \right) = g\left( {W.x + b} \right)} \cr }$$

where ${\rm \beta }$ is the connection weight vector between the ${i^{th}}$ node in the hidden layer and each node in the output layer, $h\left( x \right)$ is the output vector of the hidden layer for specific samples, $g\left( \cdot \right)$ is the activation function of Hidden layer, where W is the weight vector between the input layer and the hidden layer, b is the bias vector of the nodes in the hidden layer.

#### Model update strategy

The model update strategy plays a key role in the overall incremental learning model by determining whether to start the lightweight preprocessing model through judging the update condition to preprocess the newly generated dataset for the following efficient computation of the trained classification model, and directly affects the frequency of model updates. The model update strategy can allow the continuously generated new datasets to run on the trained classification model relatively long, thus minimizing the number of model retraining while maximizing model performance, significantly reducing time consuming and improving computational efficiency. We measure the difference degree in feature distribution between the old and new datasets based on the KL scatter value and develop the evaluation index D and design the model update strategy. The KL mathematical model is defined as follows.

(3) $$\matrix{ {D(p|{\rm |}q{\rm )} = \; \mathop \sum \nolimits_{i = 1}^n p\left( {{x_i}} \right)ln\displaystyle{{p\left( {{x_i}} \right)} \over {q\left( {{x_i}} \right)}}} \cr }.$$

We assume that each sample of the new dataset and the original dataset contains n points, considered as n-dimensional features, the calculation process of feature distribution difference evaluation index D between the new and old dataset is as follows: in the first step, we count each dimensional feature distribution of all samples in the original dataset based on equal interval bins method, detailed as follows: 1, we count maximum Max, minimum Min of each dimensional feature of all samples, then the feature value range is defined as $\Delta = Max - Min$; 2, We divide $\Delta$ equally into N intervals, then obtain the box width as $\displaystyle{{Max - Min} \over N}$; 3, Equal interval binned box statistics count each dimensional feature distribution of all samples of the original dataset as $\left[ {{f_{1old}},{\rm \; }{f_{2old}}, \cdots {f_{nold}}} \right]$; in the second step, count each dimensional feature distribution of all samples of the new dataset by the same method as $\left[ {{f_{1new}},{\rm \; }{f_{2new}}, \cdots {f_{nnew}}} \right]$; in the third step, calculate the KL scatter values of each corresponding dimensional feature of the new dataset and original datasets as $\left[ {D\left( {{f_{1old}}||{f_{1new}}} \right),D\left( {{f_{2old}}||{f_{2new}}} \right), \cdots ,D\left( {{f_{nold}}||{f_{nnew}}} \right)} \right]$; in the fourth step, calculate the mean KL value of all the corresponding dimensional features and the final evaluation index of the feature distribution difference degree between the old and new datasets is $D = {\rm \; }\displaystyle{1 \over n}\mathop \sum \nolimits_{i = 1}^n D({f_{iold}}||{f_{inew}})$. Algorithm 1 describes the model update steps.

**Algorithm 1:** The model update strategy of proposed ELM-KL-LSTM.

**Input:** Train dataset X_train; Update threshold t; Light weight model ELM;
Real time classification model LSTM.
**Output:** The updated ELM-KL-LSTM.
**Procedure:**
1: Generate a new time series dataset X_new;
2: Compute the D value between X_train and X_new;
3: If D $\ge$ the update threshold t:
run the light weight model ELM; run the real time classification model LSTM.
4: Run the real time classification model LSTM;
5: If there is another new dataset X_new: Repeat the above steps 2–4.
6: End.

We calculate the feature distribution difference degree D between the old and new datasets based on the KL scatter value. If D exceeds the update threshold t, the incremental learning model is updated by starting the lightweight model to pre-process the new dataset and then feeds the processed dataset into the efficient classification model. Otherwise, no lightweight model is started and the newly generated dataset is directly input into the efficient classification model.

#### Efficient classification model

**Figure 3:**
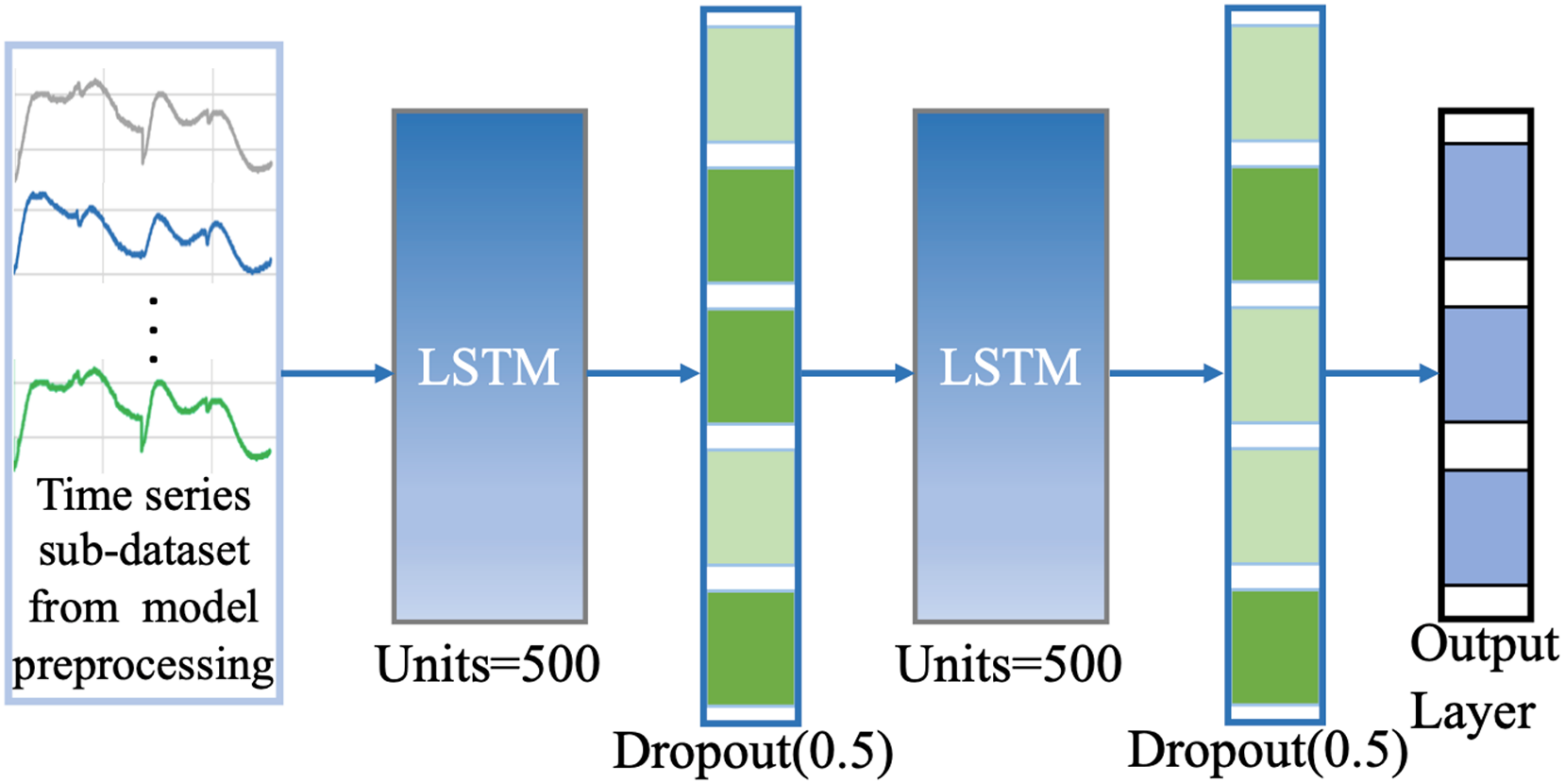
Network structure of our classification model.

Through “Model Pre-processing” and “Model Update Strategy”, we can obtain the new dataset with similar or more consistent feature distribution with the original dataset which has better and more accurate information representation, then we can directly run the trained classification model based on the original dataset to achieve efficient classification computation instead of retraining the deep classification model many times, which can reduce large time consuming and improve computational efficiency while ensuring model performance for some extent. We build the efficient classification model based on LSTM network. As a typical recurrent neural network with the characteristics of temporal memory, LSTM has a natural advantage in processing time series data and has excellent information extraction ability and robustness, and is one of the most common and powerful tools in time series models. The base network model consists of an input gate, a forgetting gate, an output gate, and a memory unit, based on the setup of the three gate structures in its internal network which can selectively extract the relevant deep nonlinear feature information in time series data. In our classification model, we design a two identical LSTM stacking model to perform efficient classification computations on the preprocessed new dataset, see Fig. 3. The first layer is the LSTM network layer, the second layer is the Dropout layer with rate = 0.5; the third layer is the LSTM network layer, the fourth layer is the Dropout layer with rate = 0.5, and the fifth layer is the Dense output layer with the activation function Softmax. The optimizer is Adam, the learning rate decay (lr) = 1e−4, The loss function loss = ‘categorical_crossentropy’, which mathematics is defined in Eq. (4). The training process evaluation matrix is classification accuracy. The other parameter Settings are detailed in Fig. 3.

(4) $${\rm L} = - \displaystyle{1 \over {\rm N}}\mathop \sum \nolimits_{\rm i} \mathop \sum \nolimits_{{\rm c} = 1}^{\rm M} {{\rm y}_{{\rm ic}}}\log \left( {{{\rm p}_{{\rm ic}}}} \right)$$

Here N denotes the sample number, M is the category number, ${y_{ic}}$ is symbolic function, ${p_{ic}}$ is the prediction probability.

## Experiments and analyses

### Datasets and data preprocessing

#### Datasets

In this article, five time series datasets in different real-world application scenarios are used, which specific description is shown in Table 1. And all have huge research value and practical significance. The class distributions for all the datasets are shown in Table 2.

**Table 1:** Datasets used in this work.

Datasets	Category	Size	Length
LIRB127	2	127	588
LIRB78	3	78	1,764
ECG	5	5,000	140
CASIA	6	1,305	28
CROP	24	31,200	48

**Table 2:** Class distributions for the datasets.

Datasets	Category	Class distributions (lab is the label of the category)					
LIRB127	2	Lab0	Lab1				
		70	57				
LIRB78	3	Lab0	Lab1	Lab2			
		26	33	19			
ECG	5	Lab0	Lab1	Lab2	Lab3	Lab4	
		2919	1767	96	194	24	
CASIA	6	Lab0	Lab1	Lab2	Lab3	Lab4	Lab5
		218	330	187	161	254	255
CROP	24	Lab0–lab23					
		1,300 samples in each category					

Plant electrical signals carry information about the response and adaptation process of environmental changes. Light induced rhythmic bioelectrogenesis (LIRB) is from wheat seedling leaf surface when under salt stress triggered by periodic light (Wang et al., 2019). As a kind of plant electrical signal, it has been proved to carry rich physiological state information, which has great research value and social significance for rapid stress resistance breeding, plant electrophysiology and the related research (Qin et al., 2020; Wang, Fan & Qian, 2018; Wang et al., 2019; Yao et al., 2021), including LIRB127 (Qin et al., 2020), LIRB78 (Wang et al., 2019) collected from different varieties of wheat seedlings with 300 mMol salt stress. ECG carries important physiological information of human beings, which is very important for judging life and health status. Detection and analysis of ECG has important physiological and medical significance. The ECG dataset in this article is from Physionet_ATM (Chen et al., 2015). The recognition and analysis of speech emotion data has always been a research hotspot, with important research significance and great difficulty in the field of automatic speech recognition (ASR). In this article, CASIA comes from the speech emotion recognition dataset of the University of Science and Technology of China. We used F-bank technique to process the audio files and the final dataset includes six categories, that is, six kinds of emotions with angry, fear, happy, netral, sad, surprise. The Crop dataset is derived from remotely sensed images from Earth observation satellites and processed into a time series dataset, which is important for efficient monitoring of the dynamics of Earth regions over time (Tan, Webb & Petitjean, 2017).

#### Data preprocessing

In order to ensure the model convergence and improve the computing speed, this article performed normalization processing for all datasets. In addition, in order to ensure the balance distribution of samples in the dataset and guarantee the model training effect, we did the shuffle processing to all samples in the dataset. The normalization definition is as follows.

(5) $$\matrix{ {{X_{new}} = \displaystyle{{X - {X_{min}}} \over {{X_{max}} - {X_{min}}}}} \cr }.$$

### Evaluation metrics

For experimental results evaluation, we chose five commonly used standard evaluation metrics: accuracy (ACC), sensitivity (SEN), specificity (SPE), F1-score (F1) and area under curve (AUC). ACC is the ratio of the number of correctly classified samples to the total number of samples (Qin et al., 2020).

(6) $$\matrix{ {ACC = \displaystyle{{TP + TN} \over {TP + TN + FP + FN}}} \cr }.$$

Herein TP, TN, FP, and FN represent the positive sample predicted by the model to be positive, the negative sample predicted by the model to be negative, the negative sample predicted by the model to be positive, and the positive sample predicted by the model to be negative, respectively (Qin et al., 2020).

SEN measures the classifier’s ability to recognize positive samples, representing the proportion of correctly classified positive samples among all positive samples (Qin et al., 2020).

(7) $$\matrix{ {SEN = \displaystyle{{TP} \over {TP + FN}}} \cr }.$$

SPE measures the classifier’s ability to recognize negative samples, representing the proportion of correctly classified negative samples among all negative samples (Qin et al., 2020).

(8) $$\matrix{ {SPE = \displaystyle{{TN} \over {FP + TN}}}}.$$

F1 provides relatively accurate evaluation for balanced and unbalanced datasets and comprehensively evaluates model performance. The higher the value, the better the model performance.

(9) $$\matrix{ {F1 = 2*\displaystyle{{precision*recall} \over {precision + recall}}}}.$$

AUC represents the probability that the score of positive samples correctly given by the model is higher than that of negative samples. The larger the value, the better the model performance.

(10) $$\matrix{ {AUC = \displaystyle{{\mathop \sum \nolimits_{i \in positive\; Class} ran{k_i} - \displaystyle{{M\left( {1 + M} \right)} \over 2}} \over {M*N}}}}.$$

Herein, $ran{k_i}\;$ denotes the number of the ith sample. M and N are the number of positive and negative samples respectively.

### Benchmarks

In order to evaluate the performance of the proposed ELM-KL-LSTM for efficient classification analysis of time series data, we compared it with several benchmark methods from the classic local learning methods such as Original LSTM (Hochreiter & Schmidhuber, 1997) and Original ELM (Liang et al., 2006) to the state-of-the-art approaches such as ActiSiamese (Malialis, Panayiotou & Polycarpou, 2022), Scikit-multiflow-HoeffdingTree (Montiel et al., 2018) and Informer (Zhou et al., 2021) through five different crucial real-world application scenarios. We conducted an extensive study and comparation analysis based on the proposed method and the benchmark methods.

### Experimental results

#### LIRB127 experimental results and evaluation

**Figure 4:**
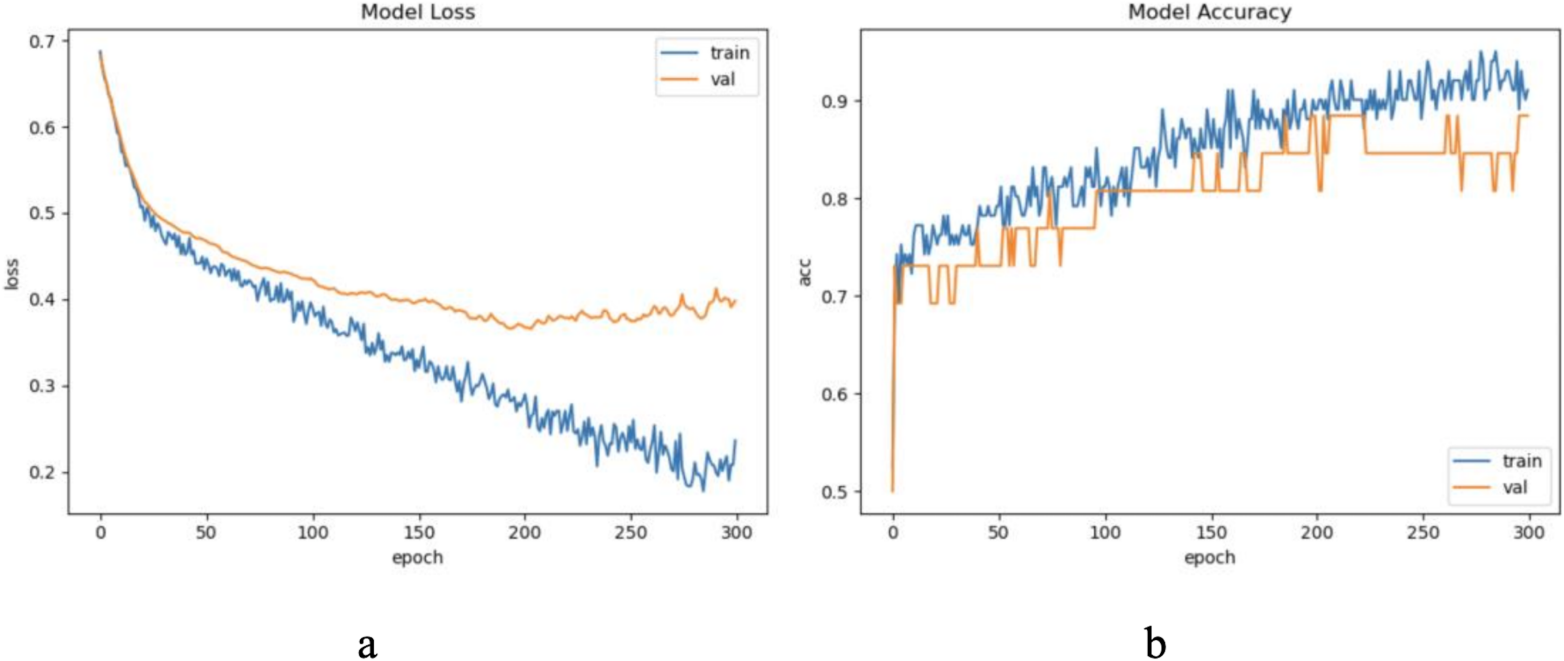
The training process of ELM-KL-LSTM. (A) The loss function change curve. (B) The accuracy change curve.

The dataset LIRB127 contains 127 samples, each waveform consists of three cycle waves, each with a wavelength of 196, the first cycle wave is from the last cycle wave before salt stimulation, while the second and third cycle waves are the first and seventh cycle waveforms after stimulation and which have been proved to carry rich plant physiological information (Qin et al., 2020; Wang, Fan & Qian, 2018; Wang et al., 2019; Yao et al., 2021). Due to salt stimulation, the second and third cycle waves have a degree of feature distribution difference and separable ability compared to the first cycle wave (Qin et al., 2020; Yao et al., 2021). Considering the periodicity and sample variability, here we used the first and second waves as training waveforms and the second and third waves as update waveforms. Therefore, in this dataset we set to update feature with an update window size of two cycle wavelengths 196 * 2 and an update step of one cycle wavelength 196, because of the limited sample length we decided the number of update windows to be 1. Through experimental exploration we set the model update threshold t = 0.60. Following the new method in “The proposed method”, we conducted experiments and evaluated the results. The model training process are shown in Fig. 4.

**Figure 5:**
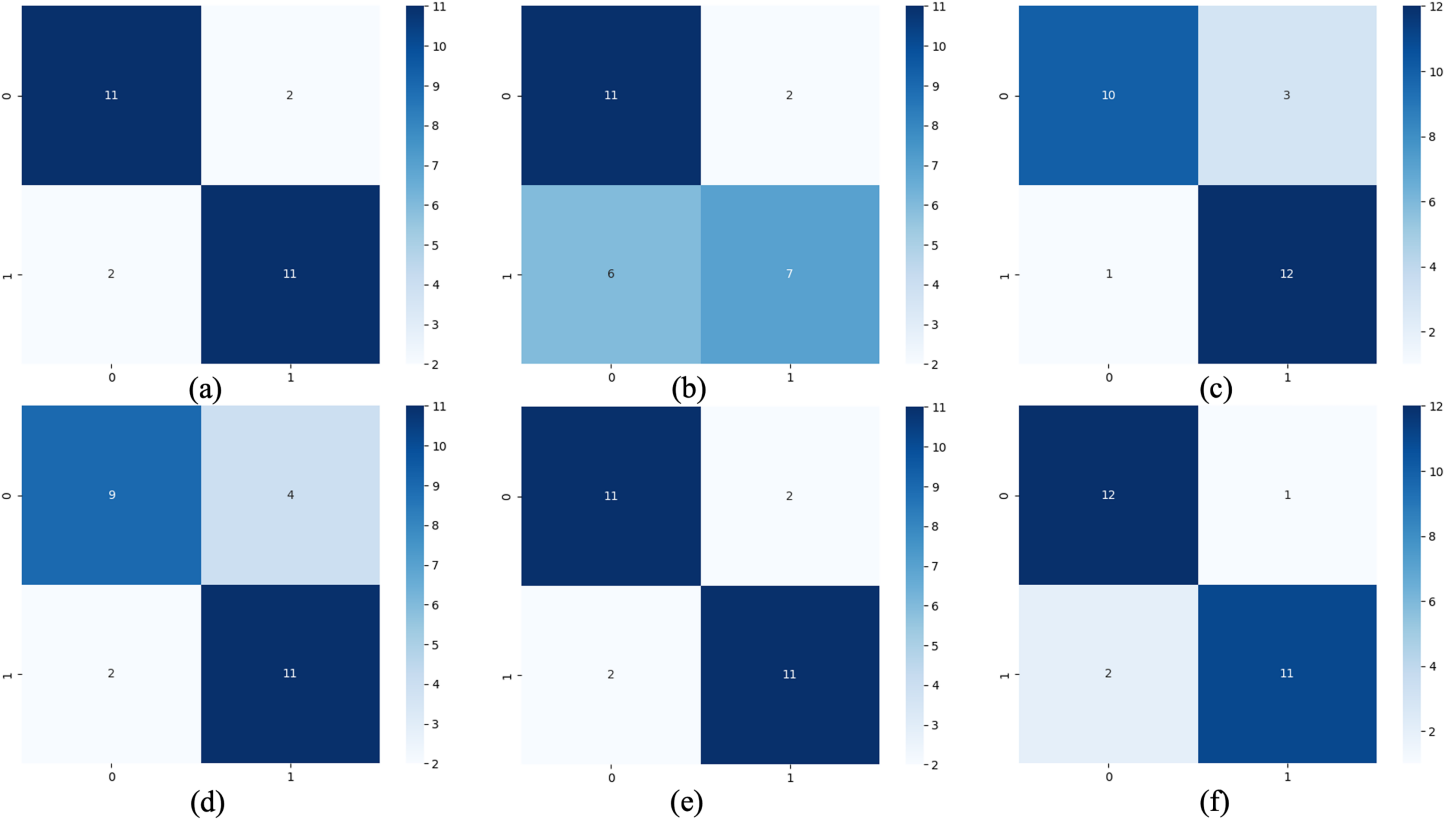
Confusion matrix of classification results. (A) The results of original LSTM. (B) The results of original ELM. (C) The results of ActiSiamese. (D) The results of Scikit-multiflow-HoeffdingTree. (E) The results of Informer. (F) The results of ELM-KL-L.

For the waveform data within the update window, we conducted experiments and comparative analysis based on the new method and benchmark methods, and evaluated the classification results for each window based on five evaluation metrics, as shown in Table 3; Fig. 5 shows the confusion matrix heat map of the classification results for each update window.

**Table 3:** Results of ELM-KL-LSTM and the comparison models.

Metrics	Original LSTM	Original ELM	ActiSiamese	Scikit-multiflow-HoeffdingTree	Informer	ELM-KL-LSTM
ACC (%)	84.62	69.23	84.62	76.92	84.62	88.46
SEN (%)	84.62	69.23	84.62	76.92	84.62	88.46
SPE (%)	84.62	69.23	84.62	76.92	84.62	88.46
F1 (%)	83.56	70.85	83.46	79.83	82.23	89.32
AUC (%)	80.23	70.21	82.57	75.12	83.34	88.43

The above results show that for the update window, the proposed method is 88.46% in ACC, SEN and SPE; the results of Original LSTM, ActiSiamese and Informer are 84.62%; the Original ELM 69.23%, and the Scikit-multiflow-HoeffdingTree 84.62%. For metrics F1 and AUC, the proposed method also outperforms the benchmarks. The comparative methods of Original LSTM and Original ELM have no preprocessing mechanism for new dataset with feature distribution difference changes, and which performance is insufficient compared with the proposed method. For the comparative methods such as ActiSiamese, Scikit-multiflow-HoeffdingTree and Informer, this is the first of which application to analyze plant electrophysiological data LIRB, while effective in dealing with ever-changing dataset, they do not show any advantages over the proposed method.

#### LIRB78 experimental results and evaluation

**Figure 6:**
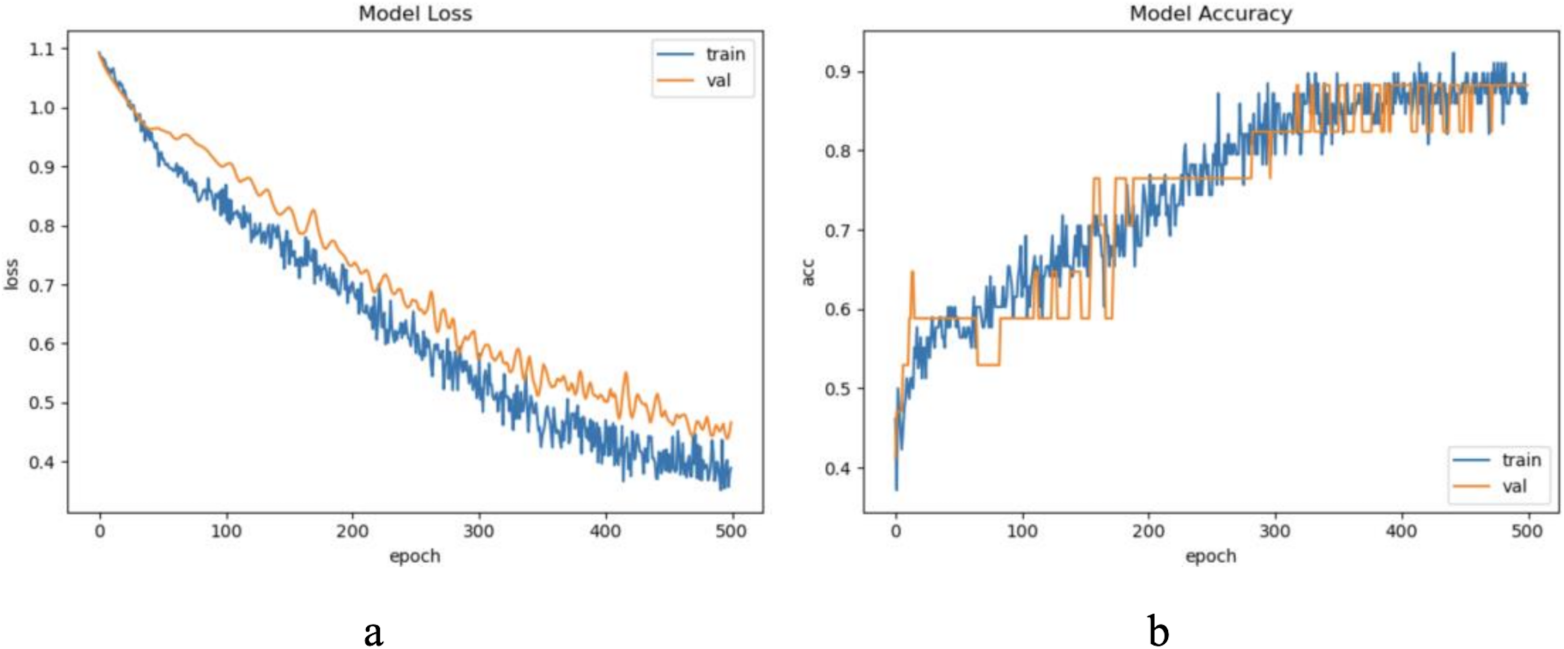
The training process of ELM-KL-LSTM. (A) The loss function change curve. (B) The accuracy change curve.

The dataset LIRB78 has 78 samples consisting of three cycle waves from three varieties of wheat seedlings with each cycle wave length of 588. Similarly, we use the first and second waves as training waves and the second and third waves as update waves. Therefore, considering the waveform periodicity and sample variability, for this dataset we set to update feature, the update window size is two cycle waveforms length 588 * 2, and the update step is one cycle waveform length 588. Due to the limited sample length we set the update window number to 1. Through experimental exploration we set the model update threshold t = 0.62. Following the proposed method in Section 3, we conducted experiments and evaluated the results. Figure 6 shows the model training process.

**Figure 7:**
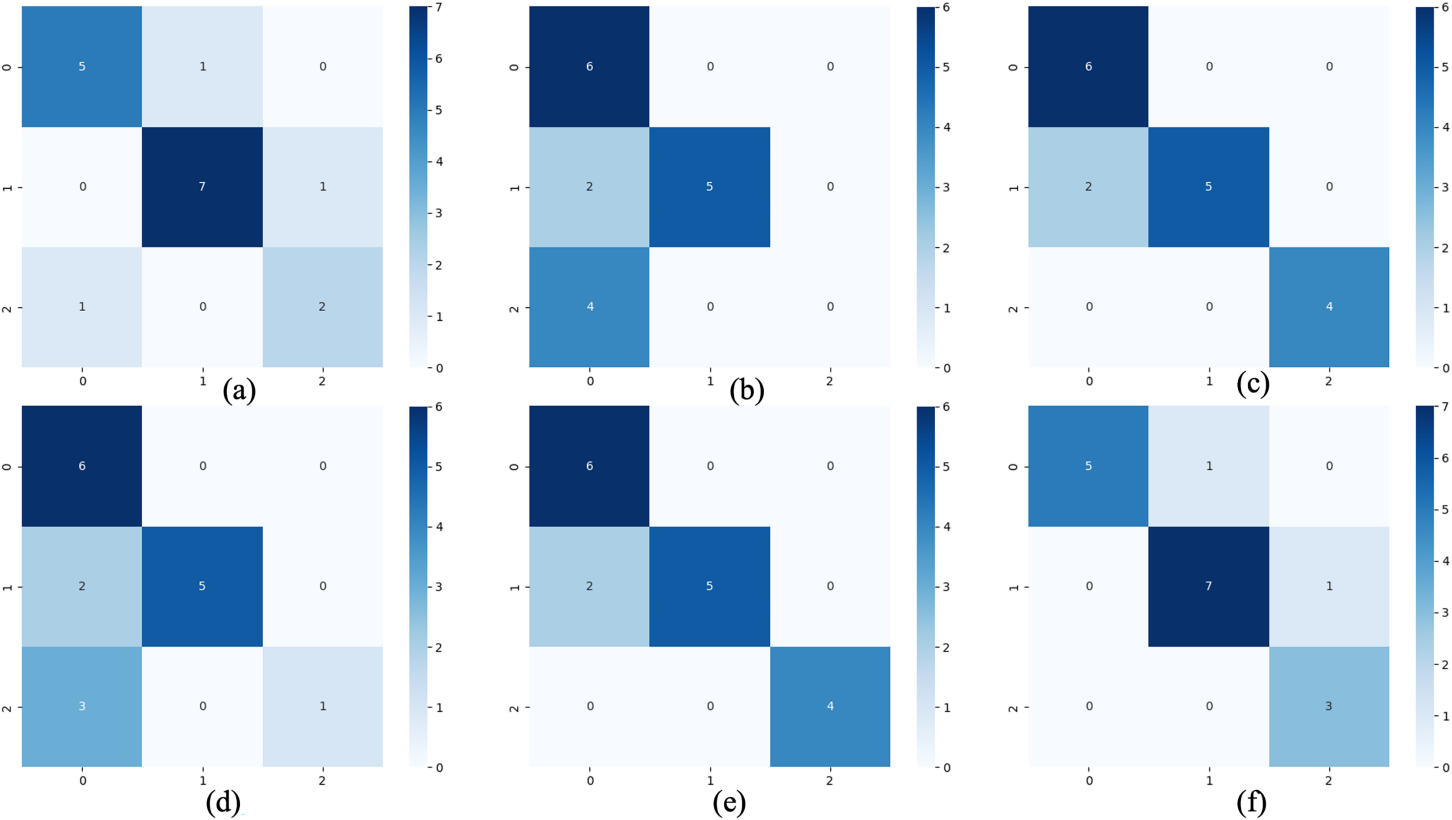
Confusion matrix of classification results. (A) The results of original LSTM. (B) The results of original ELM. (C) The results of ActiSiamese. (D) The results of Scikit-multiflow-HoeffdingTree. (E) The results of informer. (F) The results of ELM-KL-L.

For the waveform data within the update window, we conducted experimental and comparative analyses based on the proposed method and benchmark methods, and evaluated the classification results for each window based on five evaluation metrics, as shown in Table 4; Fig. 7 shows the confusion matrix heat map of the classification results for each update window.

**Table 4:** Results of ELM-KL-LSTM and the comparison models.

Metrics	Original LSTM	Original ELM	ActiSiamese	Scikit-multiflow-HoeffdingTree	Informer	ELM-KL-LSTM
ACC (%)	82.35	64.71	88.24	70.59	88.24	88.24
SEN (%)	79.17	57.14	90.48	65.48	90.48	90.28
SPE (%)	90.89	81.82	93.94	84.85	93.94	93.92
F1 (%)	80.56	66.34	89.78	78.86	89.83	89.85
AUC (%)	84.54	70.83	90.76	68.92	90.21	92.37

The above results show that for the update window, the proposed method are 88.46%, 90.28%, 93.92%, 89.85% and 92.37% in ACC, SEN, SPE, F1 and AUC respectively, having obvious improvements compared with the benchmark methods of Original LSTM, the Original ELM and Scikit-multiflow-HoeffdingTree. While the results are almost consistent with the benchmark methods of ActiSiamese and Informer. This indicates that it is very necessary to set up a reasonable preprocessing mechanism for the large variation of feature distribution difference generated by dynamic time series datasets, while the traditional methods are obviously not suitable for processing the dynamic time series datasets.

#### ECG experimental results and evaluation

**Figure 8:**
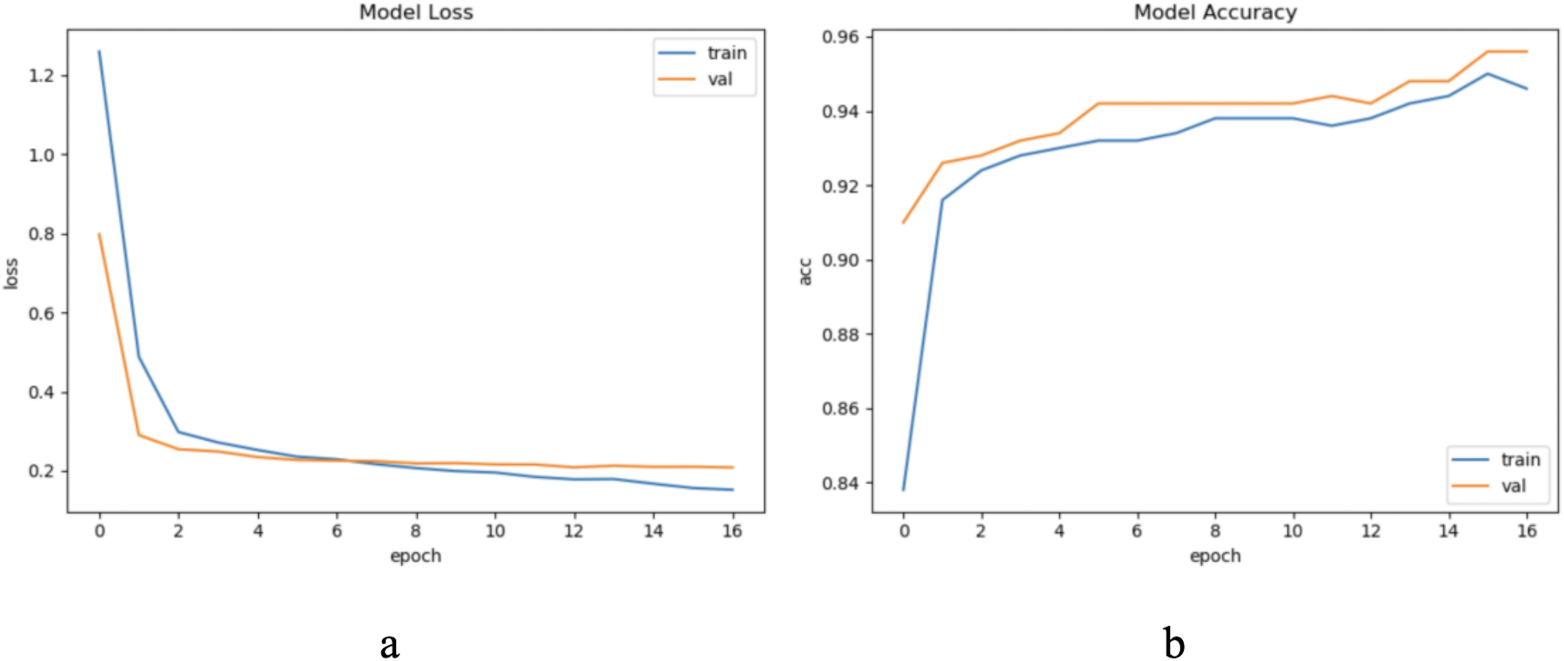
The training process of ELM-KL-LSTM. (A) The loss function change curve. (B) The accuracy change curve.

The sample length in the ECG dataset used in this article is short and the waveform does not have periodicity. To ensure the sample variability between different categories within the update window, we chose to update the sample with a window size w = 500, step = 500, and a total of five windows, through practical exploration we set the model update threshold t = 0.73. Following the proposed method in Section 3, we conducted experiments and evaluated the results. The model training process are shown in Fig. 8.

**Figure 9:**
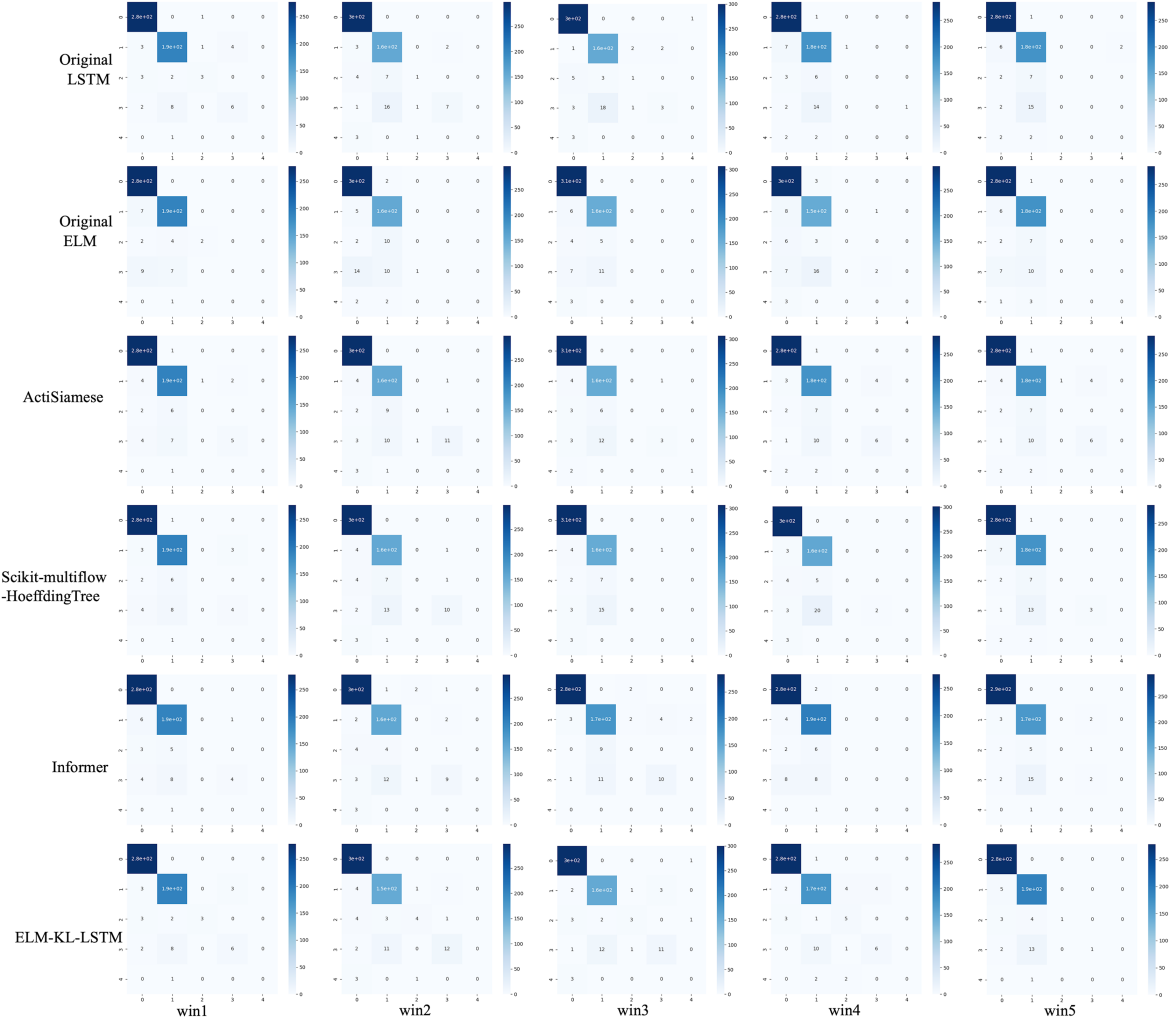
Confusion matrix of classification results of ELM-KL-LSTM model and the benchmark methods in different updated windows from win 1 to win 5.

For the waveform data within the update window, we conducted experiments and comparative analysis based on the proposed method and benchmark methods, and evaluated the classification results for each window based on five evaluation metrics, as shown in Table 5; Fig. 9 shows the confusion matrix heat map of the classification results for each update window.

**Table 5:** Results of ELM-KL-LSTM and the comparison models.

Model	Metrics	Win 1	Win 2	Win 3	Win 4	Win 5
Original LSTM	ACC (%)	95.00	92.60	94.80	92.60	92.20
	SEN (%)	54.12	46.65	52.72	43.94	39.06
	SPE (%)	98.31	97.39	97.92	97.30	97.15
	F1 (%)	92.85	90.17	92.76	90.56	90.37
	AUC (%)	93.87	90.21	93.13	90.84	90.76
Original ELM	ACC (%)	94.00	90.40	92.80	90.60	92.60
	SEN (%)	44.29	39.24	39.26	40.29	39.28
	SPE (%)	97.59	96.27	96.98	96.24	97.18
	F1 (%)	89.83	85.83	91.38	85.72	91.20
	AUC (%)	90.87	89.72	90.86	90.02	90.84
ActiSiamese	ACC (%)	94.40	93.00	93.80	93.60	93.20
	SEN (%)	45.47	48.18	49.39	46.23	46.01
	SPE (%)	97.99	97.51	97.65	97.82	97.69
	F1 (%)	91.85	90.87	91.85	91.25	91.03
	AUC (%)	93.84	91.43	92.11	92.87	92.14
Scikit-multiflow-HoeffdingTree	ACC (%)	94.40	92.80	93.00	92.40	93.00
	SEN (%)	44.32	47.38	39.39	41.23	42.70
	SPE (%)	98.01	97.39	97.41	97.21	97.42
	F1 (%)	92.14	90.35	91.42	90.35	91.15
	AUC (%)	93.84	90.23	90.73	90.13	91.92
Informer	ACC (%)	94.40	92.80	93.20	93.00	93.80
	SEN (%)	44.29	46.44	40.23	39.45	41.55
	SPE (%)	97.86	97.50	97.95	97.62	97.89
	F1 (%)	93.27	90.25	91.85	92.06	91.91
	AUC (%)	94.32	89.56	92.11	93.12	92.83
ELM-KL-LSTM	ACC (%)	95.60	93.80	94.80	94.20	94.40
	SEN (%)	54.39	55.40	52.72	54.66	57.01
	SPE (%)	98.43	97.68	97.92	97.98	98.20
	F1 (%)	93.61	90.83	92.82	92.13	92.56
	AUC (%)	94.78	92.87	93.54	93.37	93.39

**Figure 10:**
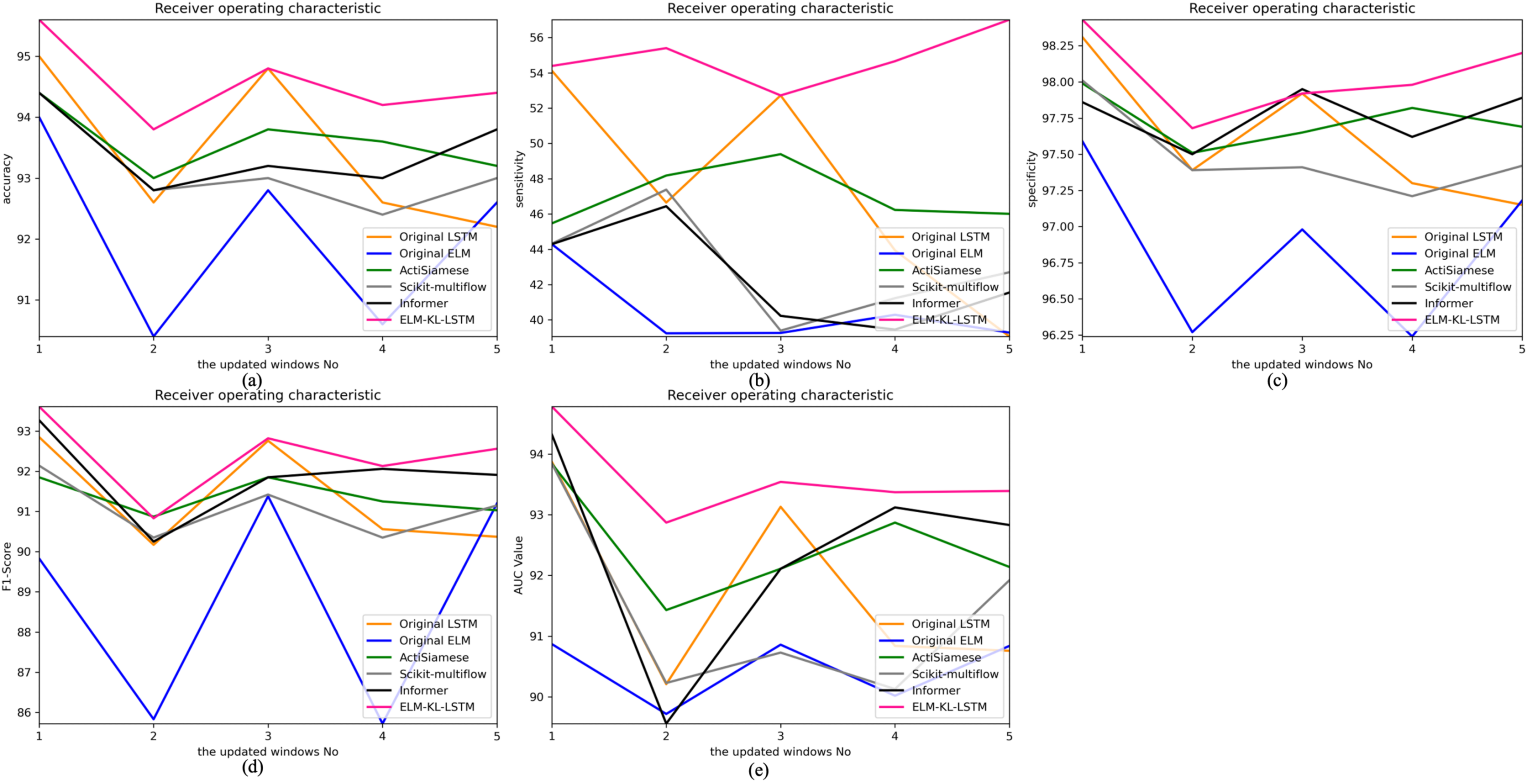
Evaluation indexes change curves of the classification results of ELM-KL-LSTM model and the benchmark methods in different updated windows from win 1 to win 5. (A) Accuracy. (B) Sensitivity. (C) Specificity. (D) F1-score. (E) AUC value.

The change curves of ACC, SEN, SPE, F1-score and AUC value for each update window are as follows in Fig. 10.

The abovementioned results show that in the five updated windows, the performance of the proposed method has been improved to different degrees compared with other benchmark methods, which proves that the proposed lightweight preprocessing model can preprocess the variation of feature distribution to some extent generated by the constantly changing new datasets. The model updating strategy based on the new designed evaluation metrics D of the difference changes of new feature distribution also ensures the effective work of the lightweight model and enables the processed data to better adapt to the original trained classification model. Therefore, to some extent it can avoid meaningless model updating, improve the generalization and training efficiency of the model, and ensure the accuracy.

#### CASIA experimental results and evaluation

**Figure 11:**
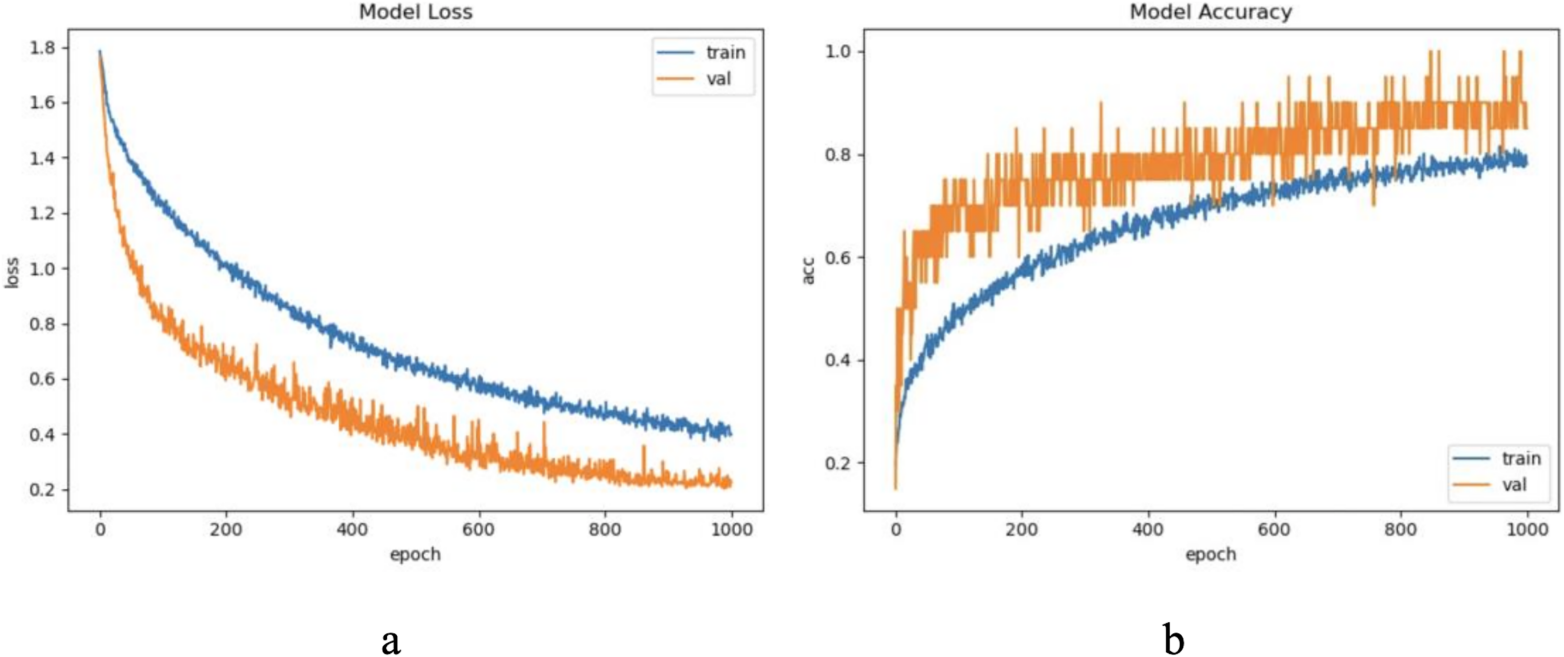
The training process of ELM-KL-LSTM. (A) The loss function change curve. (B) The accuracy change curve.

For CASIA dataset, we chose to update the samples because the sample waveform has no obvious periodicity with limited length, and set the update window size = 20, step = 20, and five update windows in total. After practical exploration we set the model update threshold t = 0.68. Following the proposed method in Section 3, we conducted experiments and evaluated the results. The model training process are shown in Fig. 11.

**Figure 12:**
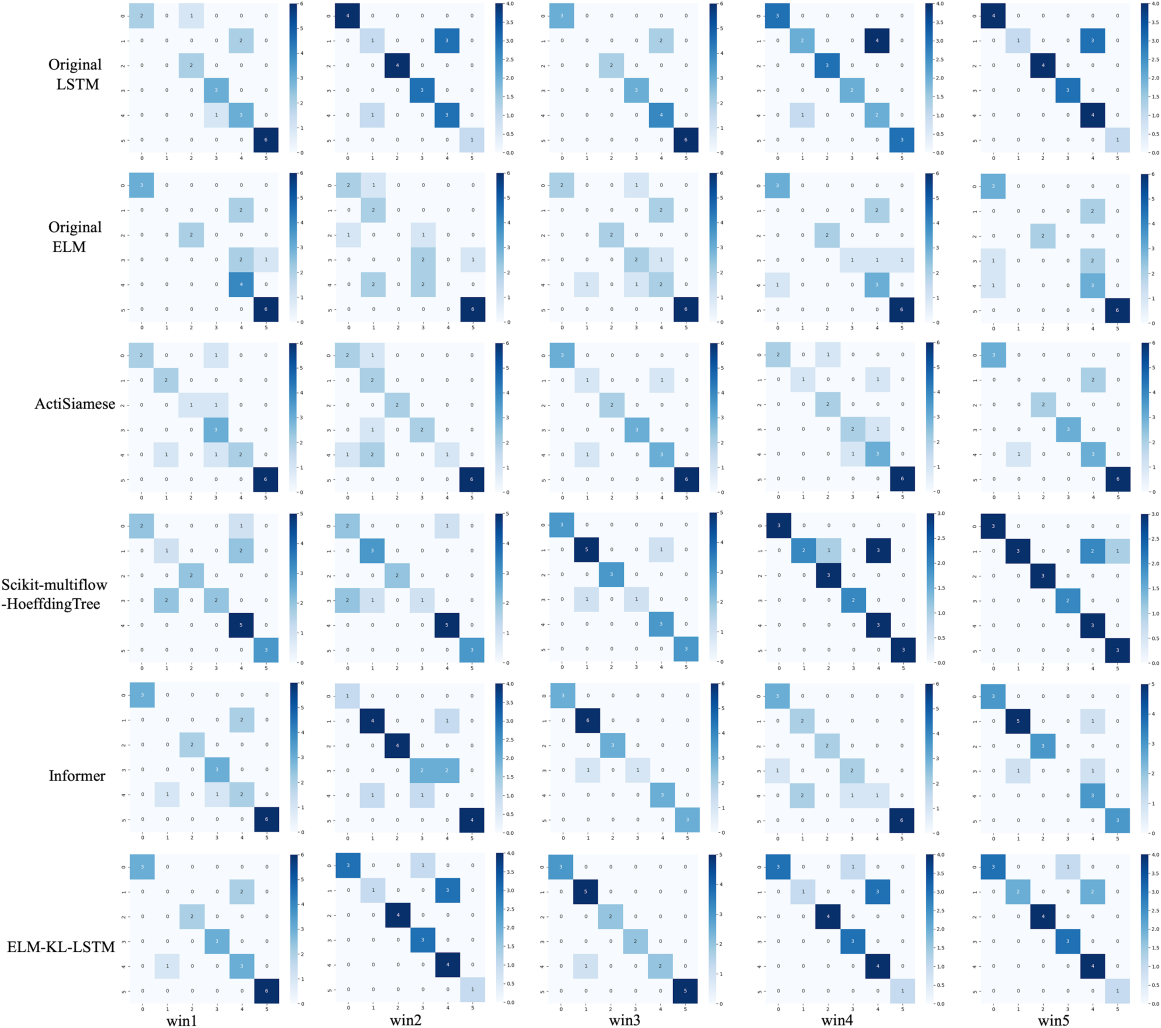
Confusion matrix of classification results of ELM-KL-LSTM model and the benchmark methods in different updated windows from win 1 to win 5.

For the waveform data within the update window, we conducted experiments and comparative analysis based on the proposed method and benchmark methods, and evaluated the classification results for each window based on five evaluation metrics, as shown in Table 6; Fig. 12 shows the confusion matrix heat map of the classification results for each update window.

**Table 6:** Results of ELM-KL-LSTM and the comparison models.

Model	Metrics	Win 1	Win 2	Win 3	Win 4	Win 5
Original LSTM	ACC (%)	80.00	80.00	91.83	75.00	85.00
	SEN (%)	73.61	83.33	83.33	79.17	87.50
	SPE (%)	96.01	95.83	97.92	94.85	96.88
	F1 (%)	88.46	84.20	90.67	80.16	89.04
	AUC (%)	89.87	88.62	92.08	82.38	90.58
Original ELM	ACC (%)	75.00	60.00	70.00	75.00	70.00
	SEN (%)	66.67	55.56	63.89	68.06	62.50
	SPE (%)	94.64	92.11	93.99	94.70	93.87
	F1 (%)	78.89	77.17	78.32	79.12	78.00
	AUC (%)	75.30	70.62	75.00	80.23	75.00
ActiSiamese	ACC (%)	80.00	75.00	90.00	80.00	85.00
	SEN (%)	77.78	76.39	87.50	76.39	79.17
	SPE (%)	96.13	95.32	98.03	96.01	96.99
	F1 (%)	88.48	80.17	90.23	84.38	89.00
	AUC (%)	89.86	83.82	90.82	85.00	89.24
Scikit-multiflow-HoeffdingTree	ACC (%)	75.00	80.00	90.00	80.00	85.00
	SEN (%)	75.00	81.94	88.89	88.89	91.67
	SPE (%)	94.71	95.95	97.83	96.08	97.06
	F1 (%)	80.32	84.00	90.30	84.43	89.13
	AUC (%)	79.76	87.43	90.78	85.12	90.62
Informer	ACC (%)	80.00	75.00	95.00	80.00	85.00
	SEN (%)	75.00	71.67	91.67	81.94	80.56
	SPE (%)	96.01	95.07	98.81	96.19	96.85
	F1 (%)	88.48	80.00	95.78	84.76	90.00
	AUC (%)	89.92	85.51	92.36	84.73	89.62
ELM-KL-LSTM	ACC (%)	85.00	81.21	95.00	82.86	84.56
	SEN (%)	79.17	88.89	94.44	83.56	85.56
	SPE (%)	96.99	96.10	98.89	95.63	96.80
	F1 (%)	88.52	84.38	95.82	86.83	90.21
	AUC (%)	90.15	88.80	92.39	85.87	89.86

**Figure 13:**
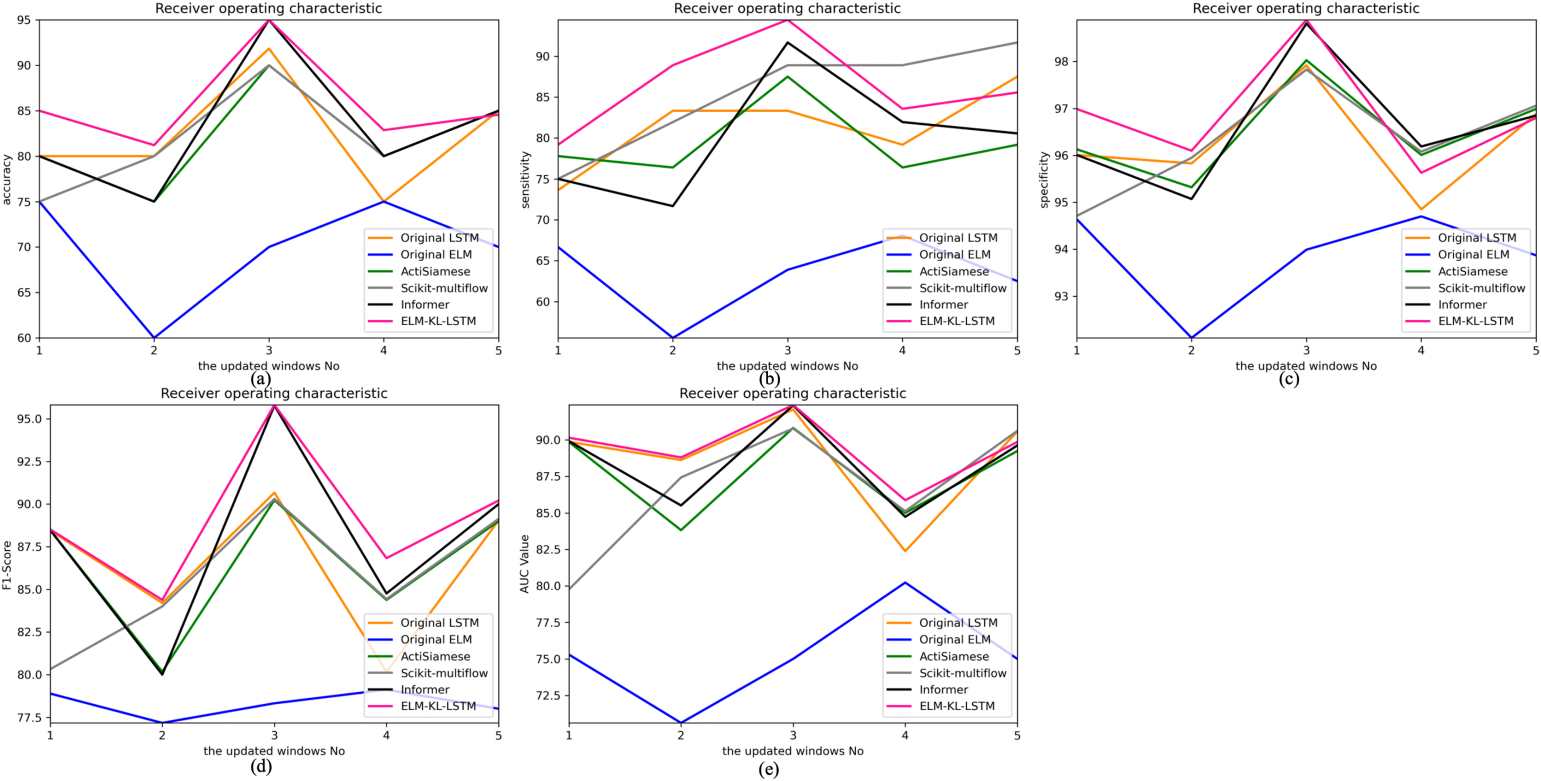
Evaluation indexes change curves of the classification results of ELM-KL-LSTM model and the benchmark methods in different updated windows from win 1 to win 5. (A) Accuracy. (B) Sensitivity. (C) Specificity. (D) F1-score. (E) AUC value.

The change curves of ACC, SEN, SPE, F1-score and AUC value for each update window are as follows in Fig. 13.

The above results show that in the first four updated windows, the proposed method has different degrees of performance improvement compared with other benchmark methods. While, in the fifth window, the performance of the proposed method is basically consistent with that of the benchmark methods such as Original LSTM, ActiSiamese, Scikit-multiflow-HoeffdingTree and Informer, which indicates that the proposed method may not have obvious preprocessing effect and performance improvement on all updated data. It also reflects that only when the feature distribution difference between the old and new datasets is too large to exceed the model update domain value, the new approach will work. The effectiveness of model updating strategy can guarantee the generalization and performance stability of the originally trained classification model to a certain extent.

#### CROP experimental results and evaluation

**Figure 14:**
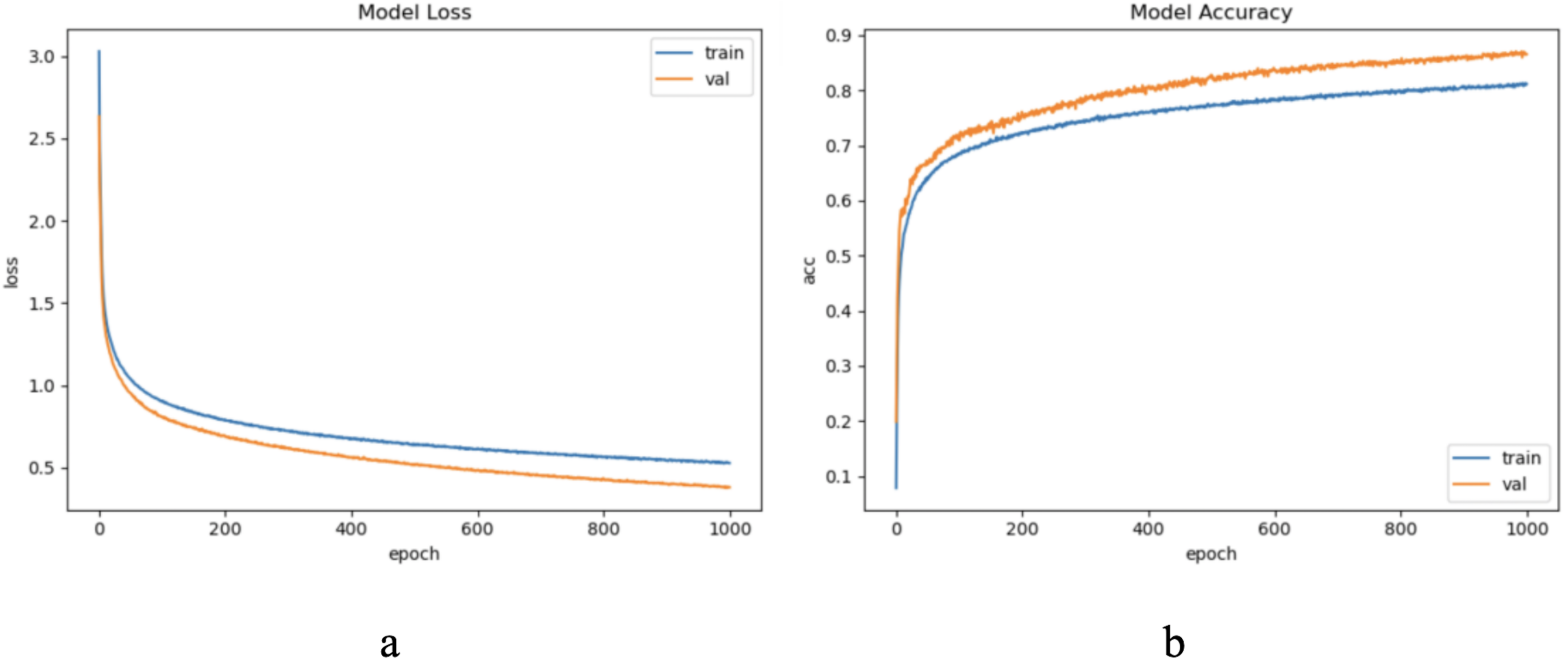
The training process of ELM-KL-LSTM. (A) The loss function change curve. (b) The accuracy change curve.

For CROP dataset, considering the sample length is limited and the waveform does not have periodicity, we update the samples and set the update window size = 1,200, step = 1,200, and a total of five windows. After practical exploration we set the model update threshold t = 0.76. Following the proposed method in Section 3, we conducted experiments and evaluated the results. The model training process are shown in Fig. 14.

**Figure 15:**
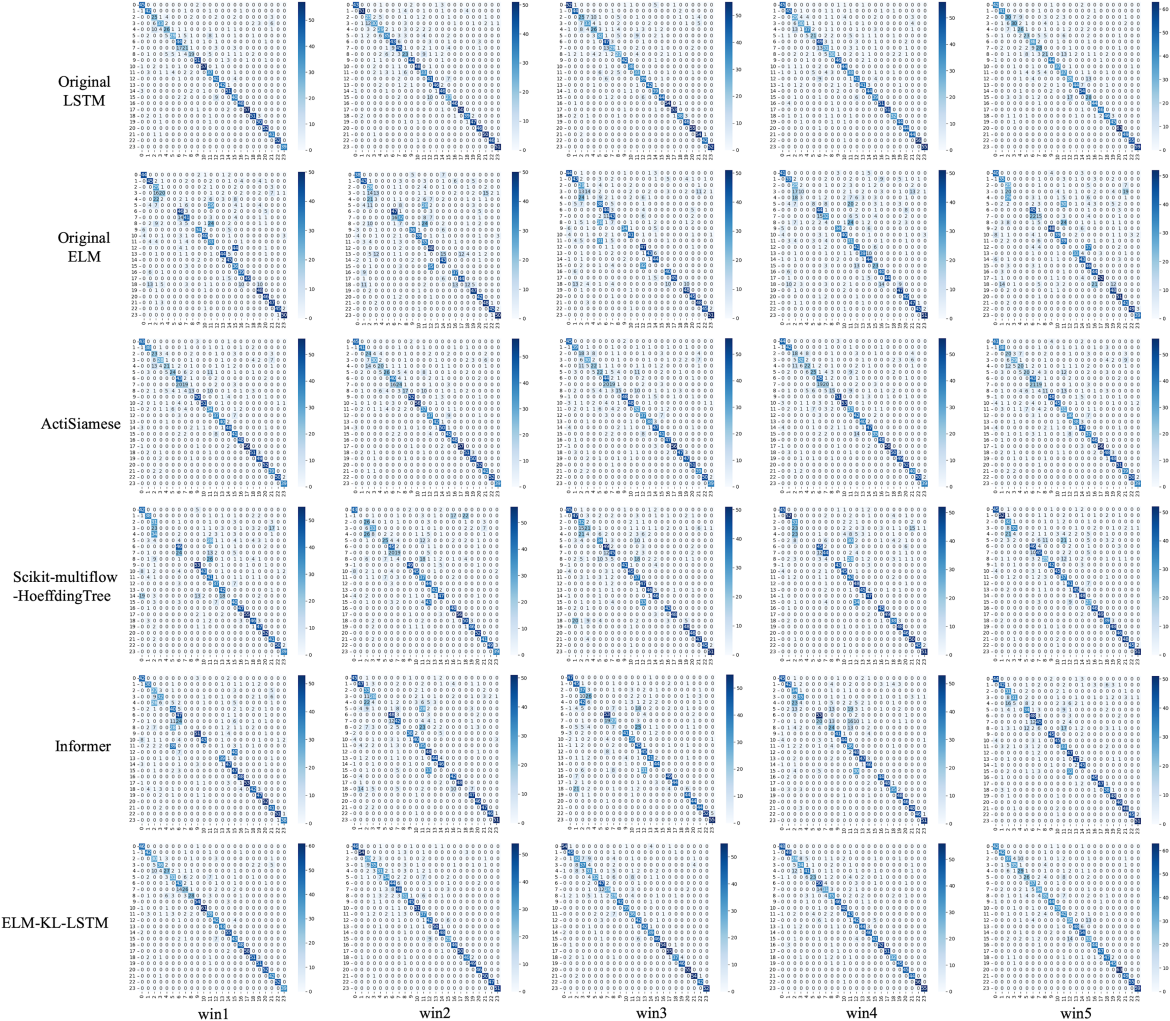
Confusion matrix of classification results of ELM-KL-LSTM model and the benchmark methods in different updated windows from win 1 to win 5.

For the waveform data within the update window, we conducted experimental and comparative analysis based on the proposed method and benchmark methods, and evaluated the classification results for each window based on five evaluation metrics, as shown in Table 7; Fig. 15 shows the confusion matrix heat map of the classification results for each update window.

**Table 7:** Results of ELM-KL-LSTM and the comparison models.

Model	Metrics	Win 1	Win 2	Win 3	Win 4	Win 5
Original LSTM	ACC (%)	82.83	82.00	82.17	82.25	80.25
	SEN (%)	82.48	82.07	82.73	82.24	80.41
	SPE (%)	99.25	99.22	99.22	99.23	99.14
	F1 (%)	85.08	86.02	86.00	86.12	84.16
	AUC (%)	86.80	85.32	86.83	85.61	82.00
Original ELM	ACC (%)	64.75	60.42	65.50	63.92	61.00
	SEN (%)	64.92	60.26	64.34	63.92	61.19
	SPE (%)	98.47	98.30	98.50	98.43	98.31
	F1 (%)	60.56	60.00	60.32	61.03	60.21
	AUC (%)	65.88	62.21	64.51	65.62	63.02
ActiSiamese	ACC (%)	78.08	81.33	76.75	79.00	75.00
	SEN (%)	77.67	80.92	76.38	78.57	74.67
	SPE (%)	99.05	99.19	98.99	99.09	98.91
	F1 (%)	80.28	85.02	79.32	82.14	79.12
	AUC (%)	79.65	80.63	80.02	80.51	77.62
Scikit-multiflow-HoeffdingTree	ACC (%)	65.83	69.58	71.92	71.42	78.08
	SEN (%)	66.16	69.13	70.93	71.22	78.41
	SPE (%)	98.52	98.68	98.78	98.76	99.05
	F1 (%)	69.21	75.39	77.39	78.00	82.51
	AUC (%)	70.91	76.82	76.90	75.02	80.18
Informer	ACC (%)	68.75	68.42	68.50	71.75	75.42
	SEN (%)	68.02	67.81	67.43	72.34	74.90
	SPE (%)	98.64	98.63	98.63	98.77	98.93
	F1 (%)	70.32	72.39	73.32	77.21	79.12
	AUC (%)	72.83	73.82	74.31	75.33	78.01
ELM-KL-LSTM	ACC (%)	86.58	86.08	84.75	85.83	85.08
	SEN (%)	86.31	86.20	85.20	85.82	85.19
	SPE (%)	99.42	99.40	99.34	99.38	99.35
	F1 (%)	90.28	90.00	88.82	90.02	89.26
	AUC (%)	89.88	89.65	90.24	90.61	90.26

**Figure 16:**
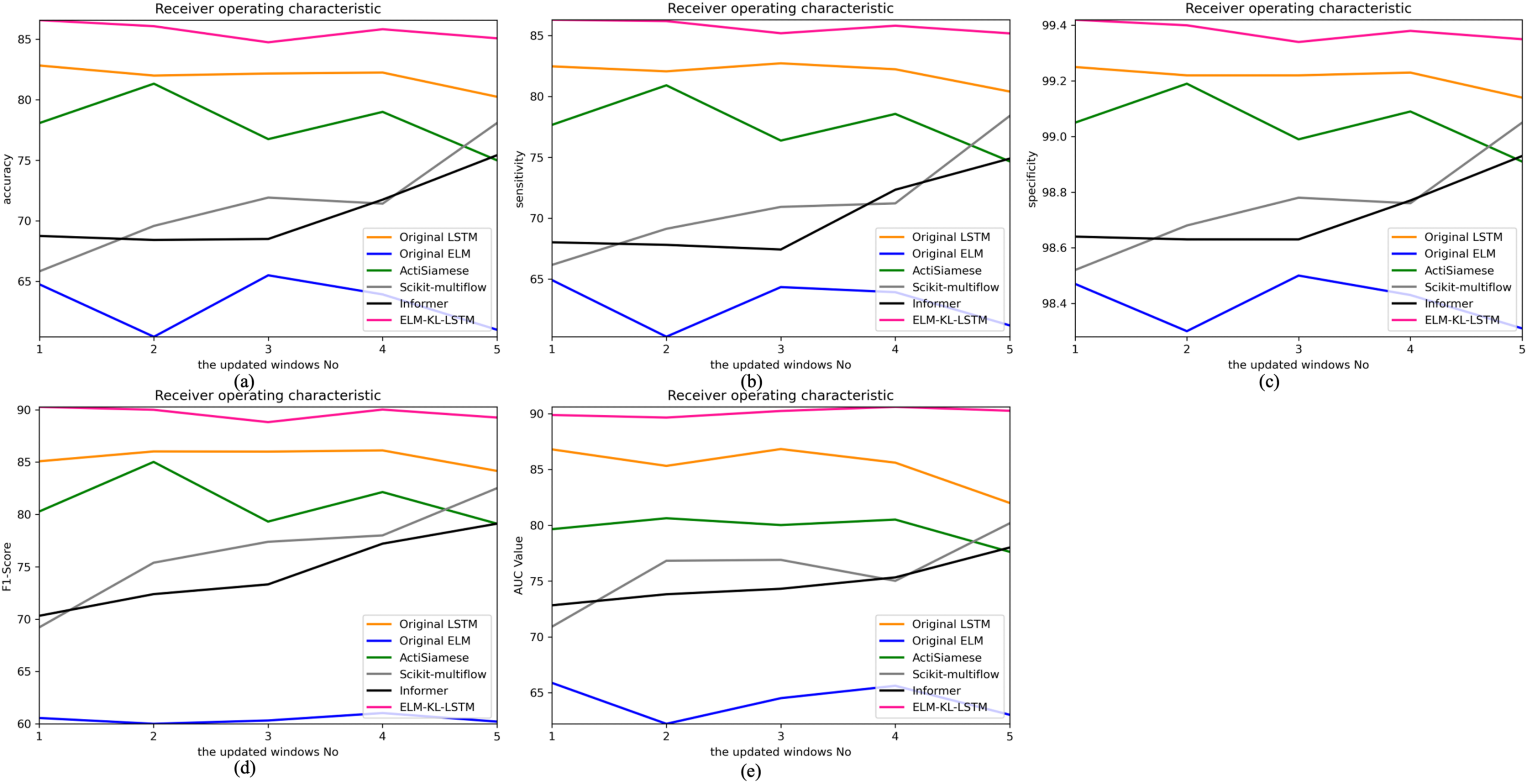
Evaluation indexes change curves of the classification results of ELM-KL-LSTM model and the benchmark methods in different updated windows from win 1 to win 5. (A) Accuracy. (B) Sensitivity. (C) Specificity. (D) F1-score. (E) AUC value.

The change curves of ACC, SEN, SPE, F1-score and AUC value for each update window are as follows in Fig. 16.

The above experimental results show that, for the five update windows in this application scenario, compared with the benchmark methods, the proposed method shows obvious performance improvement, indicating that the new designed evaluation metrics D to measure the degree of difference in feature distribution between the old and new datasets and the model update strategy based on which are effective. The lightweight preprocessing model has a certain preprocessing effect on the feature distribution changes in the new dataset, which guarantees the generalization and performance stability of the originally trained classification model to some extent.

### Discussion

#### Robustness and generalization

We validated the performance of the proposed method based on the datasets of five different real scenarios, and the results are shown in “Experimental Results”. The final classification results of the datasets within the update window in each application scenario show that the proposed method has different model updates frequencies, which proves the effectiveness, good robustness and generalization ability of the proposed model update strategy. The classification results of each scenario whenever the model is updated show that the classification results of the dataset within the update window of the proposed model are significantly improved in different degrees compared to benchmark methods such as Original LSTM (Hochreiter & Schmidhuber, 1997), Original ELM (Liang et al., 2006), ActiSiamese (Malialis, Panayiotou & Polycarpou, 2022), Scikit-multiflow-HoeffdingTree (Montiel et al., 2018) and Informer (Zhou et al., 2021). Which demonstrates the effectiveness, good robustness and generalizability of the proposed lightweight preprocessing model for multiple scenarios. Overall, the experimental results in several different real-world scenarios demonstrate its good stability, generalization and efficient classification performance of the proposed method in face of continuously updated subsets of time series data.

#### Performance differences in different application scenarios

The performance of the proposed method varies in different application scenarios, and the experimental results show that the classification accuracy of the proposed method in the update window of the plant electrical signal dataset LIRB127 is much better than that of the local learning method Original ELM, with an improvement of 11.54 percent compared to Scikit-multiflow-HoeffdingTree, and 3.84 percent compared to the other three benchmark methods. Similarly, the classification accuracy of the proposed method in the update window of dataset LIRB78 is far better than that of the classic benchmark methods Original ELM and Scikit-multiflow-HoeffdingTree, with an improving of 5.89 percent compared to Original LSTM, while the effect is consistent with ActiSiamese and Informer. The model was updated once within the update window of both plant electrical datasets, and the accuracy was also improved to different degrees, which also confirms our previous research results on plant electrical signal LIRB that which carries rich physiological state information under certain concentration of salt stress, and more importantly, has the rapid separable ability of different physiological states under different species, these have great research value and social significance for rapid stress resistance breeding, plant electrophysiology and the related research (Qin et al., 2020; Wang, Fan & Qian, 2018; Wang et al., 2019; Yao et al., 2021). The experimental results of ECG dataset shows that the classification accuracy in each update window was improved to different degrees compared to the other benchmark methods in this article, with a maximum improvement of 3.6 percent, and the model updated four times in total, and the classification accuracy was improved to different degrees in each update. For the CASIA speech dataset, the classification accuracy of the proposed method for the first four update windows have been improved to varying degrees compared to the benchmark methods. In the fifth update window, the proposed method is much better than Original ELM, while consistent with the results of the other four baseline methods. In summary, the model updated four times in total, and the classification accuracy was also improved to different degrees in each update. For CROP dataset, the model was updated in all windows and the classification accuracy had different degrees of improvement compared with the benchmark methods. In addition, the other evaluation indexes SEN, SPE, F1, and AUC are also improved in different degrees for all update windows in each application scenario. It is clear from the above that the proposed method shows good efficient classification performance in different scenarios, but its performance varies with the sample size, waveform complexity, feature dimensionality and feature distribution difference of the dataset in the update window in each application. In addition, we demonstrate the advantages, disadvantages and application scope of the methods in the existing key literature in Table 8. Experimental results show that the proposed method can effectively balance the deep information representation, calculation accuracy and effectiveness.

**Table 8:** The summarized methods of key literature.

Methods of key literature	Scope of application	Advantages	Limitations
ELM	Image processing, other one-dimensional time series data such as prediction in biology protein, climate or stocks et al.	Fast calculation speed	Weak deep information representation
LSTM	Image processing, other one-dimensional time series data such as prediction in biology protein, climate or stocks et al.	Deep spatiotemporal correlation information representation	Unable to process dynamically changing time series data efficiently
ActiSiamese	Non-stationary time series data flow	To some extent solve concept drift	Weak generalization
Scikit-multiflow-HoeffdingTree	Time series data flow processing	A certain computational efficiency	Weak deep information representation
Informer	Long time series data prediction	Deep information representation	Unable to process dynamically changing time series data efficiently
Other conventional machine learning methods	One-dimensional time series data and image processing	Good generalization	Weak deep information representation and not suit for dynamically changing time series data
Other conventional deep learning methods	One-dimensional time series data and image processing	Deep information representation	Not suit for dynamically changing time series data

#### Problems and limitations

From the experimental results, the proposed method updates the incremental learning model once in each of the two plant electrical signal dataset, four times in the ECG dataset, four times in CASIA scenario, and in the CROP scenario the model updates in each window. Although the classification accuracy is improved to different degrees, another problem is that the model update frequency is too low to improve the classification performance and too high will invariably increase the computational cost. Therefore, the study of more reasonable and efficient model update strategies and their effects on computational efficiency is a necessary work for the future. In addition, the impact of the parameters such as the size and number of update windows, update steps, and update thresholds on the efficient classification performance of the proposed method also needs to be further studied.

## Conclusion

For efficient classification analysis of dynamically changing time series data, we have proposed a new, robust, generalized incremental learning model, ELM-KL-LSTM. We conducted extensive experiments and comparation analysis based on the proposed method and benchmarks in several different real application scenarios. Experimental results show that the proposed method exhibits good robustness and generalization in a number of different real-world application scenarios, and can successfully perform model updates and efficient classification analysis of incremental data with varying degrees improvement of classification accuracy. The proposed method is suitable for the efficient analysis of dynamic time series data with large variation of feature distribution in the future, which can reduce meaningless model updating to a certain extent, improve the model training efficiency and ensure the result accuracy, as well as the performance stability and generalization of the original trained classification model, to some extent. At present, the proposed method still has some shortcomings, such as how to determine the model updating domain value is a problem, which directly determines the model updating frequency. If the model updating frequency is too high, it will affect the efficiency and increase the computation cost. However, if it too low, it will not be conducive to the effective analysis of dynamic time series data. In addition, although the single lightweight preprocessing model has a certain effect when dealing with large variation of feature distribution differences, it will be a more potential scheme if multiple lightweight preprocessing models based on ELM are integrated in the future.

## Supplemental Information

10.7717/peerj-cs.1732/supp-1 Supplemental Information 1 Code of our proposed method.

10.7717/peerj-cs.1732/supp-2 Supplemental Information 2 Five datasets we used in five real application scenarios.

## Additional Information and Declarations

### Data Availability

The following information was supplied regarding data availability: The code and raw data are available in the Supplemental Files.
